# Plasma proteome differences between giant cell arteritis and polymyalgia rheumatica: a pilot study

**DOI:** 10.1186/s13075-026-03852-4

**Published:** 2026-07-01

**Authors:** S. Seidlberger, S. Castañeda, G. Wietzorrek, K. Faserl, A. Triguero-Martínez, A. González-García, M. Schirmer, S. Santos-Sierra

**Affiliations:** 1 Institute of Pharmacology, Medical University of Innsbruck, Innsbruck, Austria; 2 Rheumatology Division, University Hospital La Princesa, Instituto de Investigación Sanitaria- Princesa, IIS-Princesa, Madrid, Spain; 3 Department of Medicine, Universidad Autónoma de Madrid (UAM), Madrid, Spain; 4 Division of Clinical Biochemistry, Medical University of Innsbruck, Innsbruck, Austria; 5 Department of Internal Medicine, University Hospital Ramon y Cajal, Madrid, Spain; 6 Department of Internal Medicine II, Medical University of Innsbruck, Innsbruck, Austria

**Keywords:** Giant cell arteritis, Polymyalgia rheumatica, Mass spectrometry, Biomarker

## Abstract

**Background:**

Giant cell arteritis (GCA) is the most prevalent vasculitis in the elderly of Caucasian ancestry, with the risk of visual loss as the most serious complication if the glucocorticoid therapy does not succeed. While imaging and temporal artery biopsy (TAB) remain diagnostic gold standards, new laboratory tests are needed to assess disease activity and follow-up monitoring.

**Objective:**

We aimed to characterize distinct proteomic signatures for the classification of polymyalgia rheumatica (PMR) and GCA (independent of concurrent therapy) and to identify specific markers of disease activity and markers that differentiate the two diseases.

**Methods:**

The plasma of 15 PMR and 13 GCA patients was analysed via mass spectrometry with the UltiMate 3000 nano-HPLC system coupled to an Orbitrap Eclipse mass spectrometer. Immunofluorescence analyses in TABs were performed to confirm the elevated plasma expression of S100A12.

**Results:**

We identified 50 protein signatures characteristic of active GCA patients, and 83 signatures altered only in active PMR samples. Strikingly, both groups shared only 13 proteins with altered protein expression levels. The newly identified proteins point to activation of biological pathways not yet linked to the diseases: mitochondrial membrane activity (ACACA, SLC25A31) and clotting cascade (VWF, TUBB) in active GCA; erythrocyte integrity (SLC4A1, SPTA1), muscle contraction (MYLK, MYL6B/12B), and glucocorticoid resistance (PTGES3) in active PMR. Importantly, S100A12 was increased not only in plasma (~ 1.6-fold), but also in PMR and GCA TABs.

**Conclusion:**

Active PMR and active GCA patients share an unexpectedly small plasma proteome signature related to immune activation (8.9%: 13 proteins). Instead, active PMR is characterized by distinct erythrocyte and muscle contraction proteome changes, whereas active GCA appears driven by mitochondrial and clotting cascade alterations. Prospective, longitudinal validation studies of the described proteomic signatures might support the clinical classification of both diseases.

**Supplementary Information:**

The online version contains supplementary material available at https://doi.org/10.1186/s13075-026-03852-4.

## Introduction

Giant cell arteritis (GCA) is an inflammatory condition of the large arteries that presents with diverse manifestations (e.g., headache, jaw claudication), with complications like blindness being the most frequent one especially if the diagnosis and appropriate treatment with glucocorticoids (GC) are not promptly attained [1]. Approximately 40–60% of GCA cases are associated with polymyalgia rheumatica (PMR), an inflammatory rheumatic disease characterised by severe muscle pain and stiffness (especially in the shoulders, neck, and hips). Also, up to 21% of PMR patients develop GCA pointing to an overlap between both diseases in what has been called the GCA-PMR Spectrum disease (GPSD) [2]. However, the factors that influence the coincidence of PMR and GCA are not well understood.

Both diseases only affect patients over 50 years of age [3]. Women present more frequently with the extracranial phenotype of GCA (EC-GCA; inflammation of the aorta, subclavian, or femoral arteries) and require more disease-modifying antirheumatic drugs. Men present more frequently a mixed phenotype, cranial (C-GCA) and EC-GCA, and show a higher mortality rate [4].

At present, the gold standards of GCA diagnosis are temporal artery biopsy (TAB) and imaging techniques using ultrasonography, magnetic resonance imaging, or the use of nuclear tracers like ^18^F-fluorodeoxyglucose. Clinical analysis of acute phase markers, such as erythrocyte sedimentation rate (ESR) and C-reactive protein (CRP) are in use, but with low specificity and selectivity [5, 6]. These markers can also be elevated during infection and other inflammatory conditions, and CRP levels are even less reliable in patients under treatment with IL-6 receptor inhibitors (e.g., tocilizumab) [7, 8]. Indeed, interleukin (IL)-6 which is considered to be a primary marker of CRP as it promotes CRP production with elevated ESR, shows higher sensitivity to diagnose active GCA than ESR. However, it is not a specific biomarker especially to differentiate between GCA and PMR particularly in the presence of bacterial infections [9, 10].

In the last years, several studies have reported potential biomarkers for prediction of disease development and the assessment of disease activity in PMR and GCA: S100 proteins [11]; pentraxin-3 (associated with recent nerve ischemia) [12]; osteopontin [13]; matrix metalloproteinases (e.g., MMP12) [14]; angiogenic modulators (e.g., VEGF, YLK-40/CHI3L1, angiopoietins) [15]; endothelin-1 (related to ischaemic complications) [16]; cytokines and related proteins (e.g., IFN-γ, IL-2, TNF-α, CCL25, CD40, CXCL9, IL-2, IL10RB, IFN-γ, MCP3 and SCF) [6, 17]; alarmins (e.g., HMGB-1, SAA, fibrinogen) [18], or altered expression of genes like *ITGA7* and *CD63* as well as genes mediating the molecular response to GC (e.g., *FKBP5*,* ADAMTS2*) [19]. However, the selection of reliable biomarkers for clinical diagnostics is proving difficult, as all these markers have rarely been directly compared in a single approach. 

We set out to analyse the plasma protein expression of active GCA and PMR patients by mass spectrometry (MS) to determine clusters and profiles of overlapping proteins that might be similarly altered in both groups of patients and also hallmark proteins that differentiate both diseases in their active state. In fact, we have found that both groups are characterised by specific proteomic signatures, but also several proteins appear commonly differently expressed in both diseases compared to normal controls. Remarkably, SAA1/2 appeared elevated in active PMR (aPMR) and SAA1 in active GCA (aGCA) samples. In contrast, mitochondrial membrane complex (ACACA, HADHA) and clotting cascade (VWF, TUBB) proteins were specifically altered in aGCA, and elliptocytosis (SLC4A1, SPTA1) and muscle contraction (MYLK, MYL6B/12B) related proteins in aPMR.

In addition, we could also determine increased expression of calcium-binding proteins of the S100 family (e.g., S100A12) not only in plasma samples, but also in TABs of PMR and GCA patients. These proteins are expressed and released at the local site of inflammation and bind to various receptors (e.g., TLR4 or RAGE) as danger-associated molecular patterns (DAMPs) [20, 21]. Taken together, these findings justify further studies to evaluate a possibly improved differentiation of GCA and PMR from other rheumatic diseases, with better disease classification and assessment of disease activity during follow-up.

## Results

### Comparative plasma proteomics of active PMR and GCA patients

The study cohort included in the proteomics analysis comprised 15 PMR patients, 13 GCA patients, and 4 controls (Table 1 and Supplementary Table 1). Active disease (aPMR/aGCA) was defined by clinical manifestations alongside elevated inflammatory markers (ESR > 40 mm/1st h and/or CRP > 0.5 mg/dL). Patients with inactive or low disease activity (iPMR/iGCA) had normal CRP values less than 0.5 mg/dl, and/or ESR values less than 30 mm/hour. The mean disease duration was 1.8 months for PMR and 26.3 months for GCA.

**Table 1:**
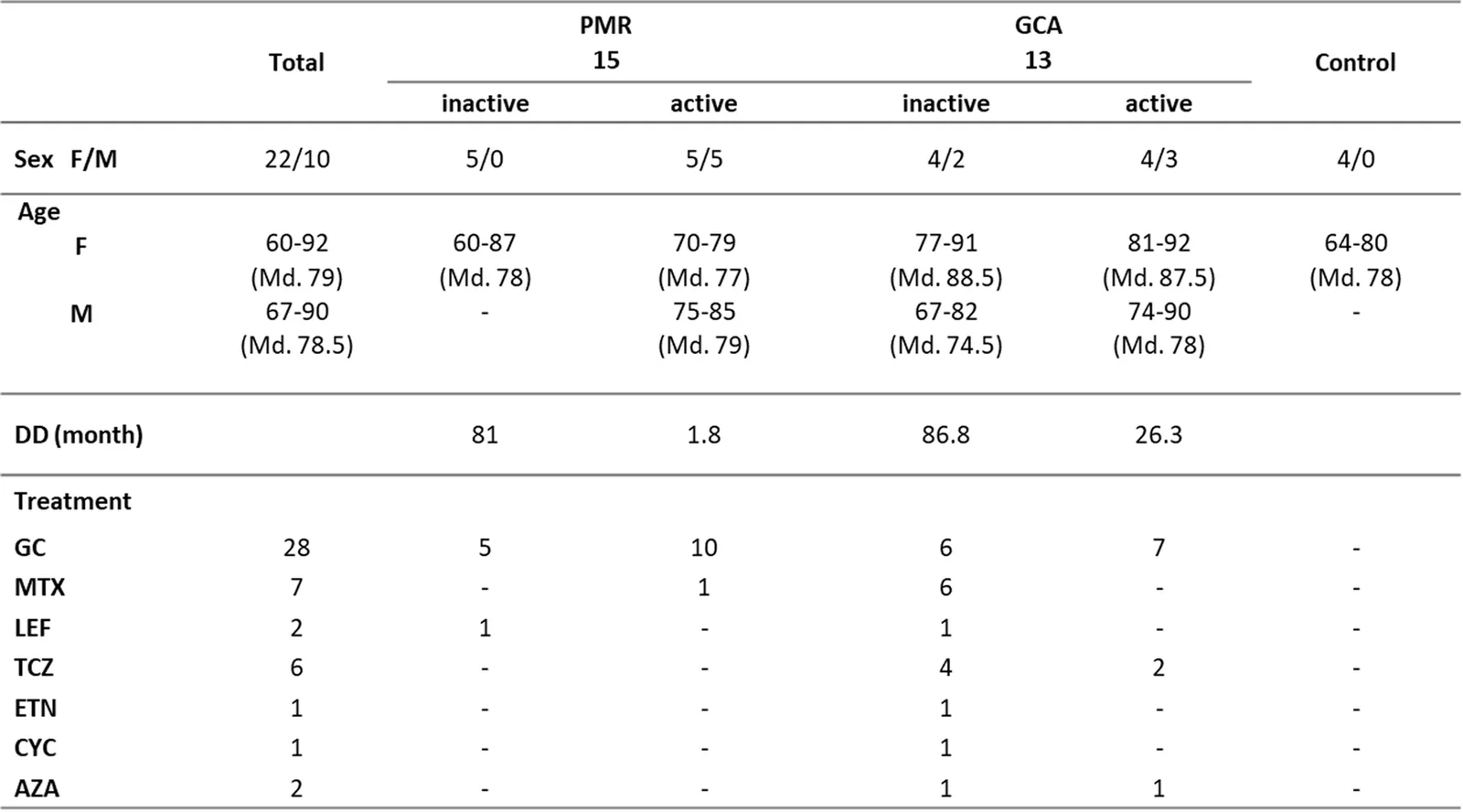
Distribution of patients in different categories and their medication

*PMR* Polymyalgia rheumatica, *GCA* Giant cell arteritis, *F* female, *M* male, *GC* glucocorticoid, *MTX* methotrexate, *LEF* leflunomide, *TCZ* tocilizumab, *ETN* etanercept, *CYC* cyclophosphamide, *AZA* azathioprine, *Md.* median, *DD* disease duration

**Fig. 1:**
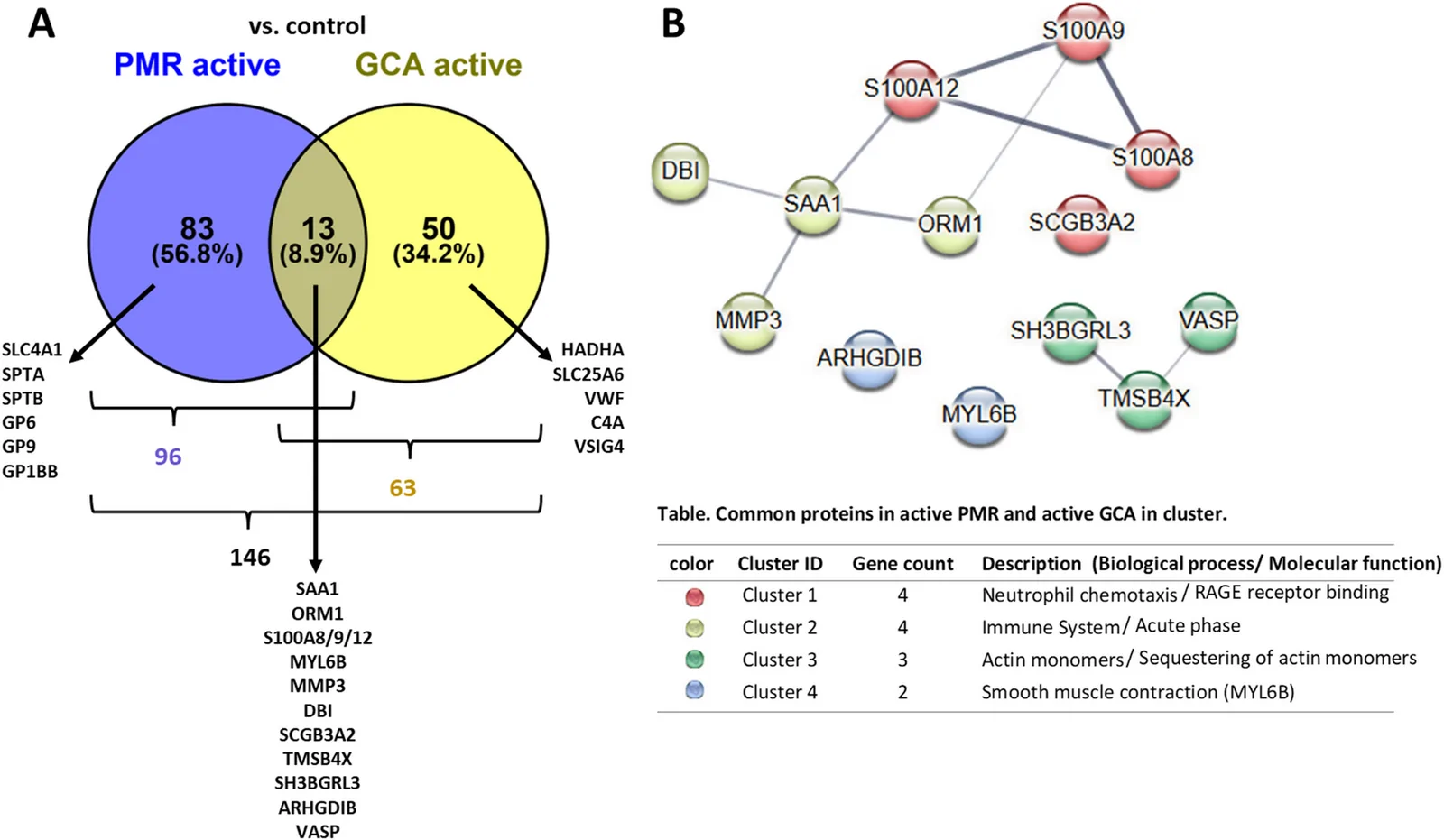
Differences in plasma proteome of active PMR and GCA patients. **A** Active PMR and GCA groups show 146 proteins differentially expressed (FC ≥ 2) to controls. The levels of 83 proteins were only altered in aPMR and 50 in aGCR. Black arrows indicate the most prominent proteins that were up- or downregulated in the respective diseases. **B** A full STRING network was constructed with the group of 13 overlapping proteins. The analysis was performed using a medium confidence threshold (0.4); the line thickness indicates the strength of data-support for the reported interactions. The table represents the identified clusters and provides a description of the STRING network. The analyses were performed with the software programs Venny (CNB-CSIC, Madrid, Spain; version 2.1.0) and STRING database (https://string-db.org/; version 12.0)

The plasma proteome of patients and controls included in this pilot study was analysed using MS. Analyses of the proteins whose expression was altered, setting a two-fold change threshold in comparison to controls, showed that 146 proteins were significantly altered in active disease states (Fig. 1A). Of these, 83 were uniquely altered in aPMR and 50 in aGCA.

Notably, only 13 altered proteins (8.9%) overlapped between the two active states. STRING functional enrichment analysis categorized these overlapping proteins into four clusters (Fig. 1B). Cluster 1 accounts proteins involved in neutrophile chemotaxis; cluster 2 includes proteins involved in immune system function; cluster 3 contains proteins related to actin monomer formation, and cluster 4 comprises 2 proteins with function in smooth muscle cell contraction. Some of these proteins have already been mentioned in other studies (e.g., SAA1, ORM1) [22], but others are newly described here (e.g., SCGB3A2 -anti-inflammatory activity-, MYL6B -myogenic regulation-).

Importantly, from the 83 altered proteins characteristic of aPMR, a set of proteins (SLC4A1, SPTA, SPTB, GP9, GP1BB) were related to spherocytosis/erythrocyte integrity. Conversely, from the 50 proteins altered exclusively in aGCA a significant group (SLC2531, HADHA) was associated with mitochondrial metabolism (Fig. 1).

### Proteomics of active versus inactive disease states

**Fig. 2:**
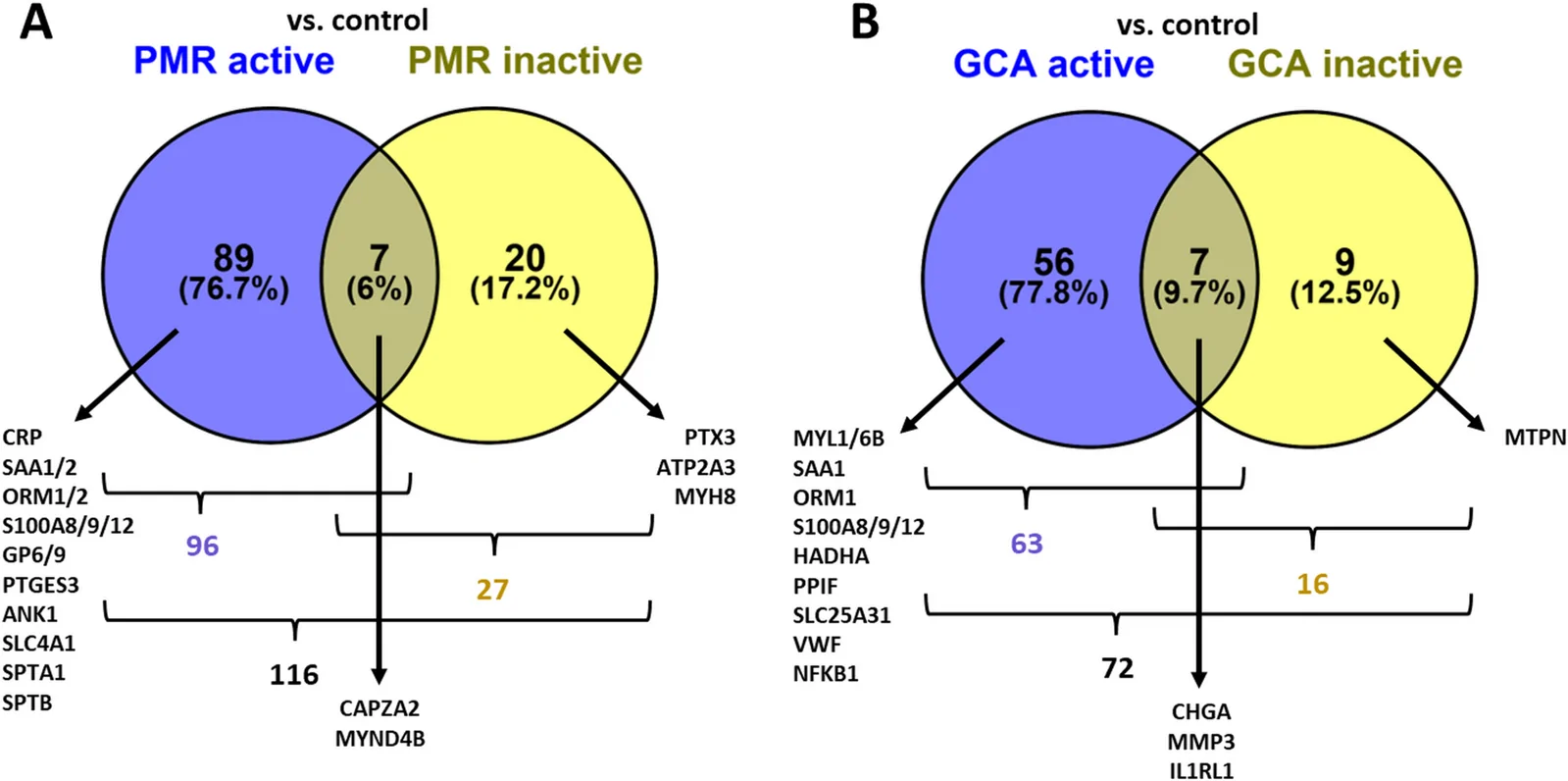
Comparison of the plasma proteome of active and inactive PMR or GCA patients. **A** Differential plasma protein expression of aPMR (*n* = 89; 76.7%) or iPMR (*n* = 20; 17.2%) patients in comparison to control samples revealed expression-altered levels for 116 proteins. Active and inactive states shared 7 (6%) common proteins. **B** Differential plasma protein expression of aGCA (*n* = 56; 77.8%) and iGCA (*n* = 9; 12.5%) in comparison to control samples, revealed expression-altered levels for 72 proteins. Active and inactive states shared 7 common proteins (9.7%). Black arrows indicate the most relevant proteins that were up- or downregulated in the respective diseases. Brackets summarize the number of proteins that exhibited a two-fold change expression (FC ≥ 2) in the analysis. All analyses were performed using the software program Venny (CNB-CSIC, Madrid, Spain; version 2.1.0)

Next, to identify signatures of disease activity, we compared active and inactive PMR and GCA patients´ cohorts against controls (Fig. 2A, B). We identified a total of 116 proteins whose abundance differed between PMR patients and controls. From those, 89 proteins (76.7%) appeared altered uniquely in aPMR samples and 20 proteins (17.2%) in inactive PMR patients, 7 proteins (6%) remained altered in both active and inactive disease stages.

For GCA plasma analysis, we identified a total amount of 72 proteins that were expressed differently from the control group. Furthermore, we detected 56 proteins (77.8%) that were changed specifically in aGCA patients and 9 (12.5%) in iGCA samples compared to the control samples. The overlap of both groups included 7 proteins (9.7%). The detailed expression profiles (significantly up- and down-regulated proteins in the plasma samples) for both cohorts, aPMR and aGCA, can be found in Supplementary Tables 2 and Table 3.

### Functional enrichment of the active PMR proteome

**Fig. 3:**
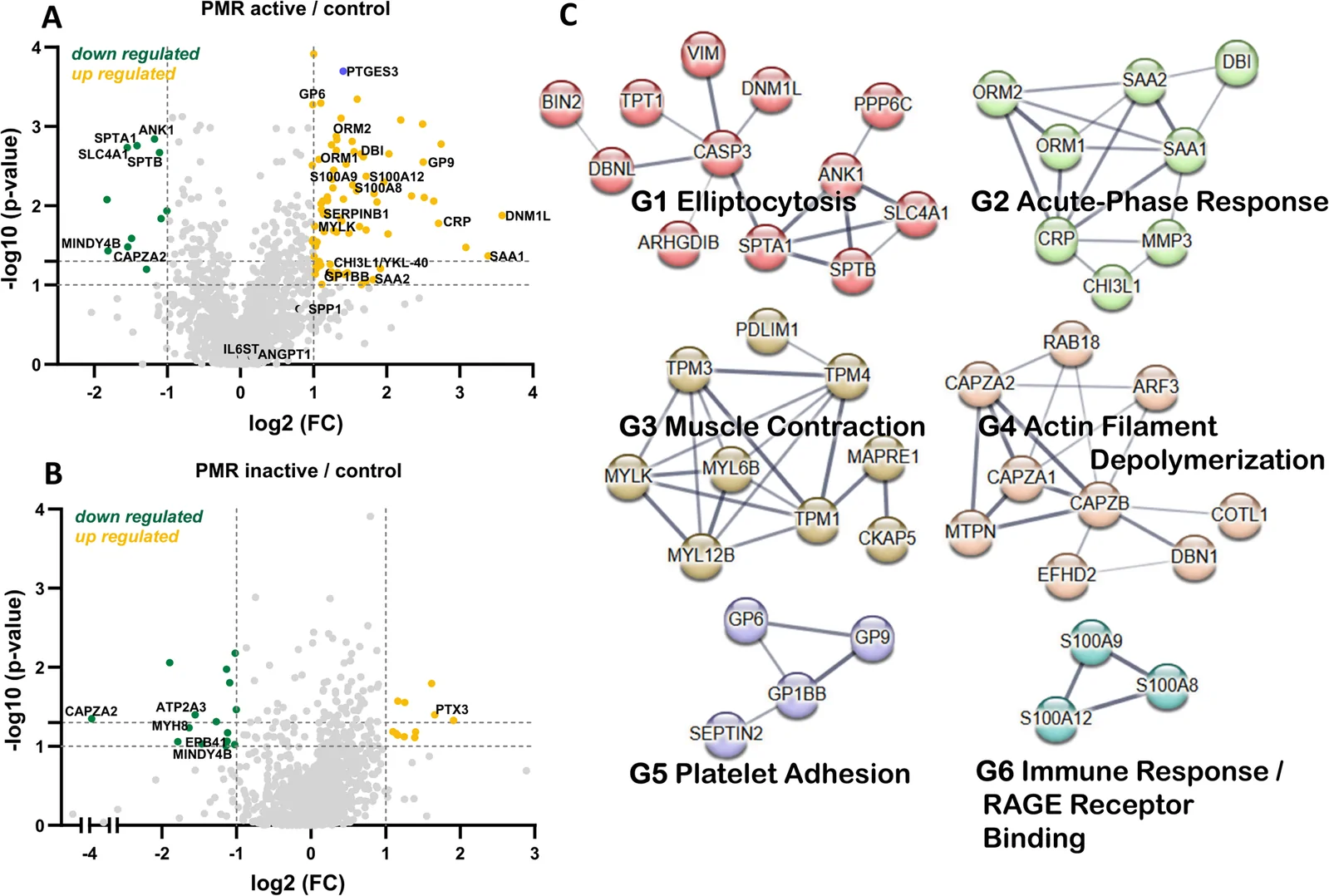
Volcano plot of up- or downregulated proteins in the plasma of PMR patients and STRING analysis. **A** Volcano plot representation of up- and downregulated plasma protein expression in aPMR samples. **B** Up- and downregulated plasma protein expression in iPMR samples. The upper quadrant in the plot represents downregulated proteins (green) and upregulated proteins (yellow) with at least two times change in expression (FC ≥ 2). The exploratory *p*-value threshold used to select proteins for STRING analysis (*p* < 0.1) and the stringent *p*-value applied for true statistical significance (*p* < 0.05), are indicated with horizontal dotted lines. Black dots are proteins described to be altered in other studies, but under the threshold levels in this analysis. The analysis was performed using GraphPad Prism 10. **C** The proteins that showed significantly altered expression levels in comparison to controls in aPMR samples were examined. The analysis was performed using a medium confidence threshold (0.4); the thickness of the connecting lines indicates the strength of data-support for the reported interactions. The clusters (G1-G6) represent putative functional pathways. The analysis was conducted using the STRING database (https://string-db.org/; version 12.0)

In order to analyse which proteins appeared altered in the plasma of PMR patients compared to controls according to the datasets obtained from MS analysis, we represented the data in volcano plots. The magnitude of protein expression change (fold change, FC) was plotted against the statistical significance of the mean differences for each protein in active and inactive PMR samples (Fig. 3A, B). We focused our analyses on proteins with a stringent absolute FC of at least two in reference to control samples. In the analysis, 84 proteins showed an upregulation in expression levels, while 12 proteins appeared downregulated. The most relevant proteins are shown in Fig. 3A.

In aPMR samples, significant elevations were found in acute phase reactants and prothrombotic factors (e.g., CRP, SAA1/2, ORM1/2, S100A8/A9/A12, GP6/9). Of note, PTGES3 (prostaglandin E synthase) emerged as a highly significant marker of active PMR. In contrast, in iPMR, PTX3 appeared significantly upregulated, and S100A10/12, although elevated, fell below the two-fold change threshold.

In both PMR phases, a downregulation of CAPZ2 (actin polymerization) and MYND4B (deubiquinase activity) was observed.

Proteins that were further downregulated in aPMR samples in relation to controls were ANK1, SLC4A1, SPTA1, and SPTB (involved in erythrocyte formation and elliptocytosis), and in iPMR, ATP2A3 and MYH8 (muscle contraction). The complete dataset of up- and down-regulated proteins that were detected at *p*-values lower than 0.1 in active PMR plasma samples in comparison to controls is listed in Supplementary Tables 2 and Table 3.

The pool of proteins with a FC expression higher than 2 and that appeared significantly deregulated in aPMR samples (*n* = 96) was included in a STRING analysis in order to retrieve signalling pathways related to the disease (Fig. 3C). The analysis retrieved six functional clusters of proteins with relevant biological activities (as determined by the number of included nodes). In the largest group (group 1 -G1-), proteins associated with erythrocyte membrane stability were found (e.g., SLC4A1, SPTA1, SPTB, ANK1). In G2 proteins involved in the acute-phase response (e.g., CRP, ORM1/ORM2, MMP3) were identified. G3 comprised proteins responsible for myocyte contraction (e.g., MYLK, MYL6B, MYL12B). G4 included proteins involved in actin filament depolymerisation (e.g., CAPZA1/2, CAPZB, MTPN). In G5, proteins that are involved in platelet adhesion (e.g., GP6/9/1BB) were grouped. G6 corresponded to proteins involved in the immune response (e.g., S100A8/9/12). Table 2 summarizes the main protein groups from the STRING analysis that are up- or down-regulated in aPMR patients compared to controls.

**Table 2:**
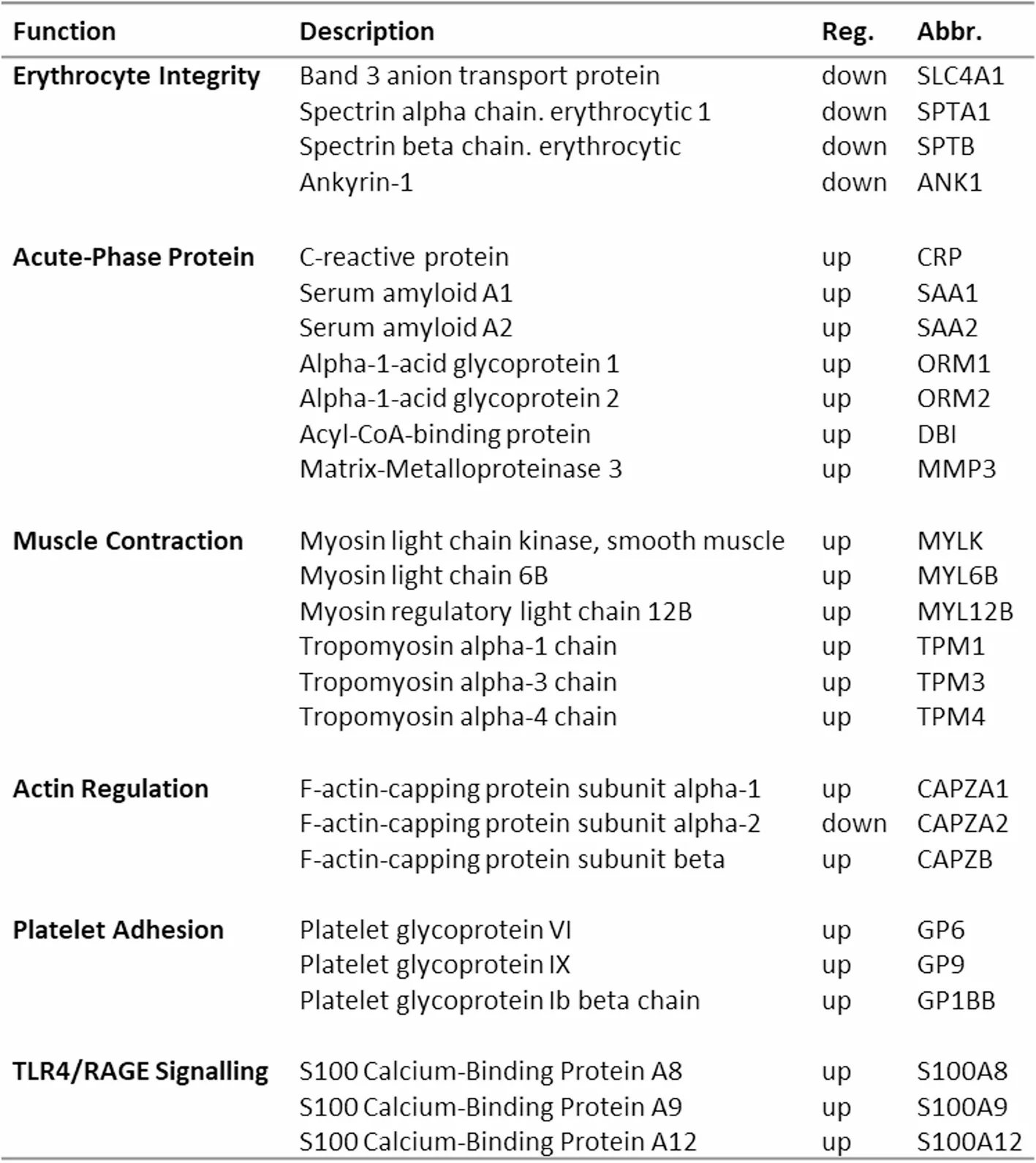
Proteins up- or down-regulated in active PMR patient plasma

### Functional enrichment of the active GCA proteome

**Fig. 4:**
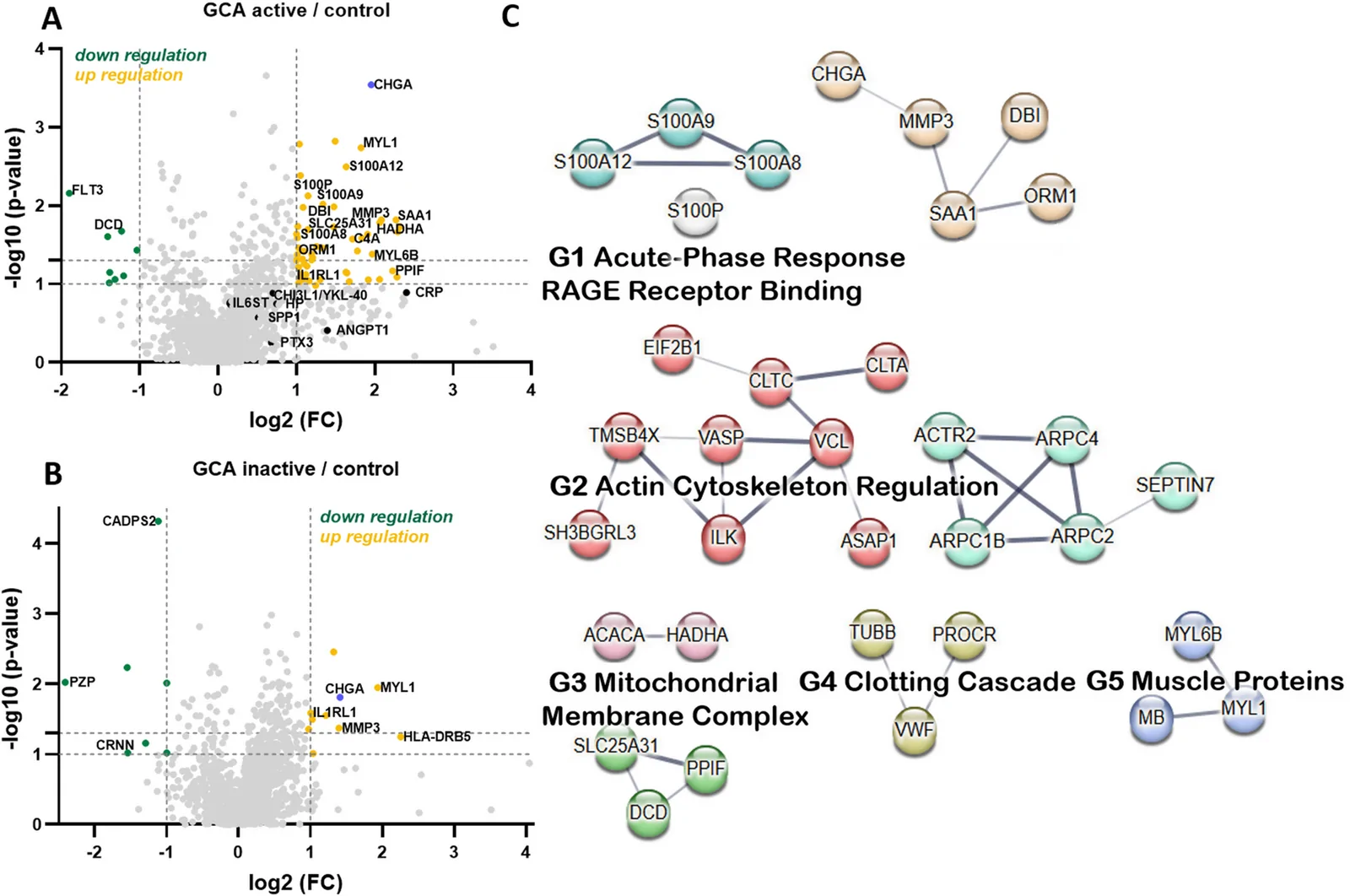
Volcano plot of up- or downregulated proteins in the plasma of GCA patients and STRING analysis. **A** Volcano plot representation of up- and downregulated plasma protein expression in aGCA samples. **B** Up- and downregulated plasma protein expression in iGCA samples. The upper quadrant in the plot represents downregulated proteins (green) and upregulated proteins (yellow) with at least two times change in expression (FC ≥ 2). The exploratory *p*-value threshold used to select proteins for STRING analysis (*p* < 0.1) and the stringent *p*-value applied for true statistical significance (*p* < 0.05), are indicated with horizontal dotted lines. Black dots are proteins described to be altered in other studies, but under the threshold levels in this analysis. **C** The proteins that showed significantly altered expression levels in comparison to controls in aGCA samples were examined. The analysis was performed using a medium confidence threshold (0.4); the thickness of the connecting lines indicates the strength of data-support for the reported interactions. The clusters (G1-G5) represent putative functional pathways

The volcano plot representation of the protein expression change in relation to the confidence value of the expression-change in plasma samples of GCA patients compared to controls is shown in Fig. 4A, B.

In aGCA samples, the MS analysis detected upregulated proteins involved in the acute phase response (e.g., SAA1, ORM1, S100A8/A9/A12); mitochondrial proteins (e.g., HADHA, SLC25A31, PPIF), and muscle proteins (e.g., MYL1/6B). In contrast, other previously described altered proteins in GCA, like CRP or CH3L1/YKL-40, did not appear over the threshold of two-fold expression changed in comparison with controls. Remarkably, IL6 did not appeared significantly elevated.

In both GCA groups (active and inactive forms), proteins involved in interleukin-1 receptor activity (IL1RL1) and the matrix metalloproteinase activity (MMP3) appeared upregulated. Interestingly, CHGA (chromogranin A) and MYL1, also appeared strongly upregulated in both GCA forms. In addition, in inactive GCA samples, MTPN (myotrophin), which promotes the growth of cardiomyocytes (cardiac muscle hypertrophy), was also elevated.

In order to define the biological processes modulated by the described proteins, we included those whose levels were significantly altered (*n* = 63) in a STRING analysis. Using this method, several biological processes were identified which are differentially activated in aGCA patients in comparison with controls (Fig. 4C; Table 3).

**Table 3:**
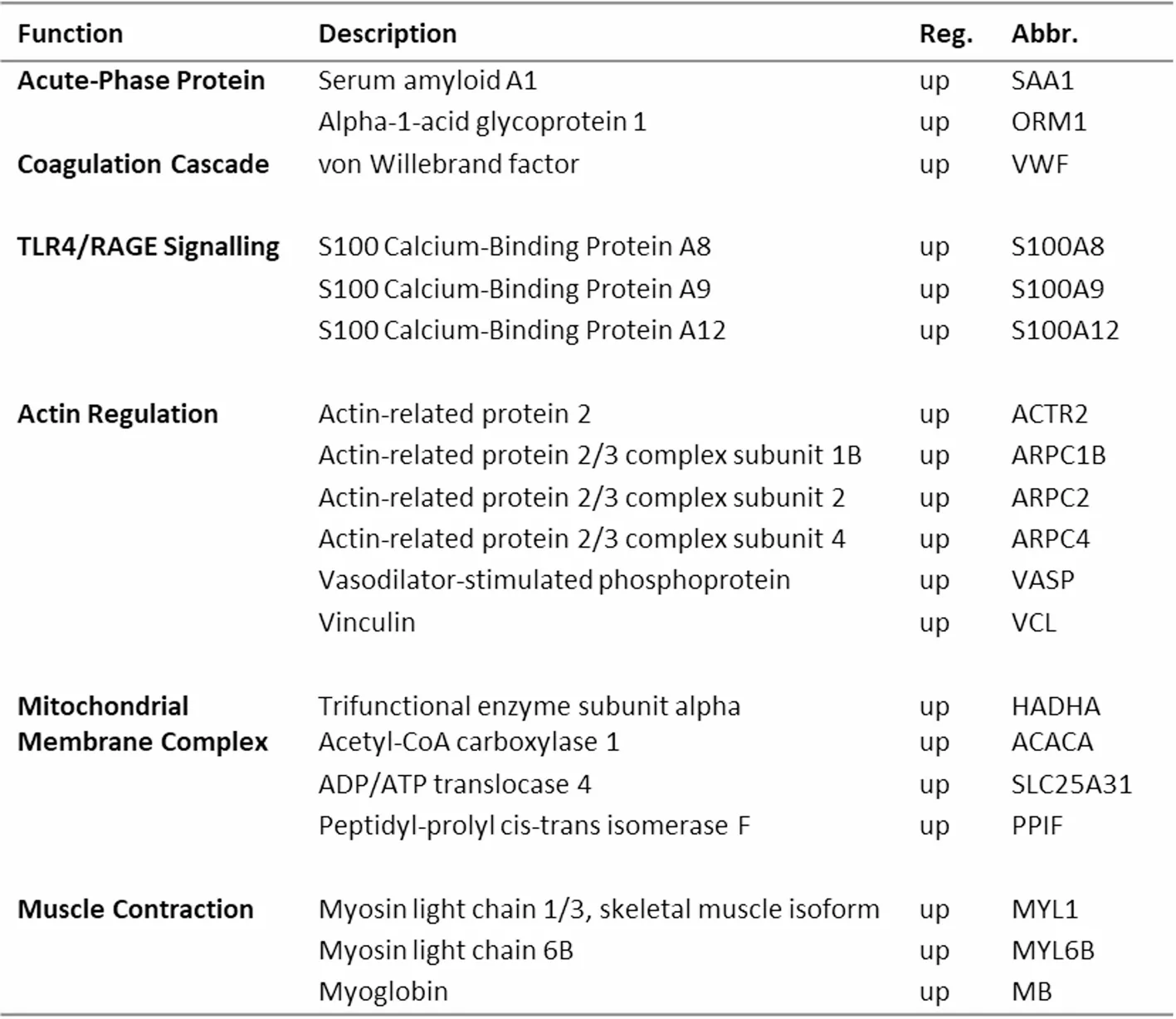
Proteins up- or down-regulated in active GCA patient plasma

Based on the STRING analysis, five groups with different biological activities were retrieved, from which we inspected the most relevant proteins in detail. In G1, we identified proteins from the acute-phase response and RAGE-receptor binding (e.g., SAA1, ORM1, S100A/8/9/12). In G2, we identified proteins involved in actin cytoskeleton regulation (e.g., ARPC2, ARPC4, ARPC1B, ILK, TMSB4X). G3 corresponded to mitochondrial proteins (e.g., HADHA, PPIF, SLC25A31). G4 clusters proteins of the coagulation cascade (e.g., VWF, PROCR), and G5 muscle-related proteins (e.g., MYL1/6B).

Table 3 summarizes the major protein groups from the STRING analysis that are up- or down-regulated in active GCA patients compared to controls. The full dataset of proteins that were up- or down-regulated in active GCA plasma samples compared to controls, with *p*-values below 0.1, is provided in Supplementary Table 3.

### S100A12 is overexpressed in temporal artery biopsies of GCA-patients

S100A12 plays a role in the regulation of inflammatory processes and the immune response (e.g., recruitment of leukocytes and promotion of cytokine and chemokine production), and it acts as an alarmin via RAGE.

**Fig. 5:**
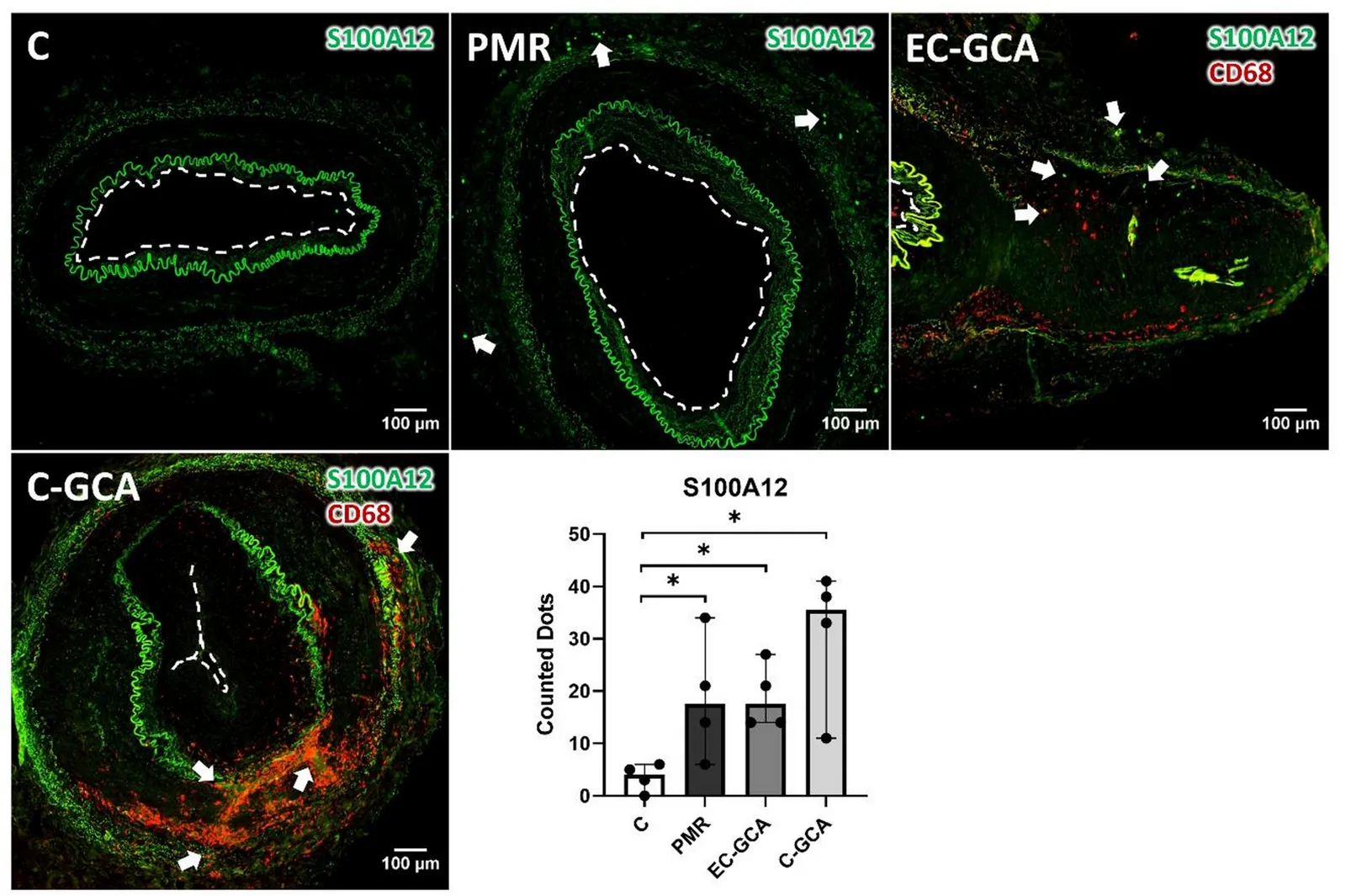
Expression of S100A12 in TABs from PMR, EC-GCA, C-GCA patients, and controls (*n* = 4). S100A12 protein expression in TABs was assessed by immunofluorescence staining. In addition, CD68 staining was performed in extracranial GCA (EC-GCA) and cranial GCA (C-GCA) samples to identify macrophages within the tissue. Quantitative analysis was conducted using a point-counting approach with ImageJ software (FijiWin64/ImageJ2015). Arrows indicate TAB regions with elevated S100A12 expression. Bar graphs display the median values and standard deviation obtained from four independent patient samples. Statistical significance was evaluated using the Mann-Whitney U test (*p* < 0.05 (*)). For clarity, the arterial lumen is delineated by an open white line. C, control. PMR, Polymyalgia rheumatica. EC-GCA, extracranial GCA. C-GCA, cranial GCA

According to the previous MS results, S100A12 expression in inactive PMR and GCA samples was similar to controls, however, in active PMR and GCA, it appeared more than 3-times elevated. Thus, we investigated whether this elevated expression might also be observed in TABs of PMR and GCA patients. To this end, we performed immunofluorescence studies of PMR, C-GCA, EC-GCA and control TABs (patients’ description in Supplementary Table 4) stained with anti-S100A12. The calcium-binding protein appeared significantly overexpressed in the media and adventitia of C-GCA samples, where also macrophages were localized (CD68 expression) but also in PMR and EC-GCA, in contrast to controls (Fig. 5).

## Discussion

We performed a proteomic analysis of plasma samples from active GCA and PMR patients to better understand the commonalities and differences between the two related diseases. Strikingly, we found less than 9% overlap in differentially expressed proteins between the two conditions. This suggest that although GCA and PMR frequently coexist, they might be driven by distinct molecular programs. Nevertheless, as shown in Tables 2 and 3, both active diseases share alterations in key functional pathways (e.g., acute phase proteins, muscle contraction, actin regulation, and TLR4/RAGE signalling). These shared pathway modifications, despite involving divergent proteomic profiles, may stem from potentially cofounding factors such as variations in time since diagnosis, prior medication use, or underlying comorbidities. However, they may reflect a fundamental baseline immune response characteristic of inflammation. Consequently, incorporating control groups with other inflammatory conditions in future analyses would be essential to validate these findings.

Some of the proteins with altered expression characterized in our study are a confirmation of previous published works, however, others have not been described in this context yet.

Earlier reports sought to identify serum markers for GCA and PMR diagnosis, or disease-activity monitoring, and to understand the impact of different treatments (e.g., GCs) [19, 23]. Interleukin-6, CRP, and ESR are considered the most important, clinically stable, and most widely used biomarkers for PMR and GCA diagnosis and monitoring. However, they are not specific and drug therapies (e.g., tocilizumab/IL-6R inhibitors) can alter their levels, rendering them ineffective for classifying the stage of the disease [24]. A retrospective study, in which the association between CRP and ESR with IL-6 was investigated in PMR and GCA patients, showed that IL-6 has a lower correlation factor with CRP or ESR than the two biomarkers among each other [18].

In previous studies, acute inflammatory proteins appeared commonly elevated in GCA, as indicators of an ongoing systemic inflammation (e.g., CRP, SAA, haptoglobin) [16, 25]. Also, cytokines and chemokines levels were altered in the serum of GCA patients (e.g., IL-6, IFN-γ, MCP-3, IL-2, and MIF) [12], pointing to activation of T-cells, monocytes, and macrophages. Due to vessel-wall damage and remodelling, proteases were also altered (e.g., MMP1/2/12) [26]. As stated, some of the above-mentioned proteins appeared also altered in our study in both active diseases, such as acute phase proteins (SAA1, ORM1). However, this cluster was also populated with proteins related to neutrophil chemotaxis (S100A8/9/12, SCGB3A2) and smooth muscle contraction and remodelling (MYL6B). In previous studies, the ratio ANGPT2/ANGPT1, together with low MMP-3, was predictive of GCA in PMR patients [15]. We did not detect ANGPT2, although ANGPT1 and several related proteins (ANGPTL1/3/4/7/6/8) were detected, but were not significantly increased in comparison to controls.

The most significant finding in the aPMR cohort was the upregulation of PTGES3. Beyond its role in basal Prostaglandin E2 (PGE2) synthesis, which contributes to the pain and morning stiffness that define the clinical presentation of PMR [27], PTGES3 serves as a co-chaperone for the glucocorticoid receptor [28]. We speculate whether the failure in GC receptor folding might affect its functionality and be related to the PMR relapses during tapering or the non-responsiveness of some patients to standard GC doses.

We observed a significant downregulation of SLC4A1, SPTA1, SPTB, and ANK1, proteins related to erythrocyte integrity, in aPMR. Since visual inspection of the specified plasma samples indicated that hemolysis during sample preparation was not the cause for our finding, it is plausible that high systemic IL-6 levels in PMR might suppress erythroid precursors and increase red blood cell fragility. Furthermore, the elevation of GP9 might reflect the intense systemic inflammatory state, whereas the persistent elevation of PTX3 in inactive PMR might indicate a smoldering inflammatory baseline even in clinically quiescent patients.

Further, clusters of proteins related to activities already described in aPMR appeared also in our study. For example, high levels of acute phase proteins (e.g., CRP, ORM1/2, MMP3, SAA1/2, PTGES3) and proteins involved in the immune response (e.g., S100A8/9/12) and in muscle contraction (e.g., MYLK, MYL6B).

In aGCA, the levels of some proteins involved in the inflammatory response appeared altered (CHGA -chromograninA-, S100A8/9/12). CHGA is a pro-hormone typically associated with neuroendocrine signaling, but it also serves as a precursor to catestatin (a peptide with potent vasoactive and anti-inflammatory properties) [29]. CHGA is also stored in endothelial and smooth muscle cells, and its elevation in plasma likely reflects neovascularization and the tissue repair processes following immune-mediated arterial damage. The fact that CHGA remained elevated in inactive GCA patients simultaneously to MYL1 (myosin light chain 1, smooth muscle layer) might reflect the ongoing vascular remodelling or chronic endothelial dysfunction rather than acute-phase systemic inflammation.

Most remarkably, in aGCA, we found enrichment of mitochondrial proteins such as SLC25A1 (mitochondrial citrate carrier) or HADHA (Hydroxyacyl-CoA Dehydrogenase, beta acid oxidation). It is likely that a metabolic “switch” fuels the inflammatory behaviour of T-cells and macrophages. The overexpression of SLC25A1 might support the metabolic demand of the inflamed arterial wall by exporting citrate into the cytosol to support the synthesis of pro-inflammatory prostaglandins, cytokines, and fatty acids. An alternative hypothesis arises from the studies of Michailidou et al. [30], which show that GCA patients have impaired ability to clear extracellular mitochondria, and this may contribute to inflammation and platelet activation. However, the cellular origin of these components in plasma is unknown. It has been described that activated platelets extrude mitochondria that can contribute to inflammation through DAMPs (e.g., oxidized mitochondrial DNA, N-formylmethionine) [31, 32, 33]. Michailidou et al. [34] found calprotectin (S100A8) and fMET peptides elevated in LVV and AAV, and fMET seemed to propagate neutrophil activation via release of calprotectin from neutrophils.

In addition, our study is the first to confirm that S100A12 is significantly elevated not only in plasma [35] but also within the temporal artery biopsies of PMR, EC-GCA, and C-GCA patients. Its ability to function as an alarmin binding RAGE and TLR2/4 and to inhibit matrix metalloproteinases (MMP2/3/9) via zinc chelation suggests a complex role in modulating the tissue remodelling that follows vascular injury [36].

The observed differences between our results and previous reports might be due to several factors: complexity of the samples, sample preparation for MS analysis, technical variations in the MS instrumentation (e.g., we used a high resolution, last generation spectrometer), and data analysis (processing of raw spectral data and bioinformatic approaches). In addition, we restricted our analysis to proteins with substantial changes in expression (absolute FC of at least two) compared to control samples. For example, different from us, a recent report by Cunningham et al. [23] did not find large differences between active and inactive GCA patients. Instead, a higher number of proteins differentiating inactive GCA from controls (e.g., B2M, C9, CD8A), suggesting persistent immunomodulation even during remission, rather than just transient inflammation. In our study, while the active groups presented approximately 75% of the proteome abundance altered in relation to controls, the inactive groups showed very low differences to control samples (iPMR 17.2% and iGCA 12.5%).

Additionally, some proteins described to be up-regulated in previous studies, appeared in our volcano plots below the significance range or fold-change threshold defined as reliable (e.g., IL6, SPP1 -osteopontin-, HP -haptoglobin-, ANGPT1) [12]. The reason for this discrepancy might be explained because some high-abundance proteins are depleted during the sample preparation step for mass spectrometry (e.g., haptoglobin). In turn, it allows the detection of proteins otherwise obscured by the very high abundant ones.

The main limitation of our study is the small cohort size. Therefore, it cannot be excluded that comorbidities and the use of different medications may influence the results. Moreover, elevated plasma levels may reflect compensatory responses to the medications. Finally, not all altered proteins might be specific to GCA or PMR if not, their levels many reflect general inflammation or immune activation. Thus, although this study provides promising results, there is a definite need for larger, independent cohorts, including disease controls (other inflammatory disorders) to assess the specificity of our findings.

In conclusion, the reports studying gene-modifications [19, 37] and variations in protein expression in different conditions (e.g., active versus inactive patients, different therapies) have not rendered definitive answers to the existence of a GCA- or PMR-specific biomarker so far. According to the results in our study, we propose that the levels of distinct proteins might be specifically upregulated in GCA (proteins related to mitochondrial integrity) and other specifically downregulated in PMR (proteins related to elliptocytosis). Additionally, we hypothesize whether modulating PTGES3 might overcome steroid resistance in PMR and provide steroid-sparing alternatives for patients who are refractory to conventional treatments.

## Materials and methods

### Plasma isolation and cytokine measurement

Plasma samples were obtained from 32 patients. Following separation of blood cells using Histopaque^®^-1077 from 18 ml of whole blood, plasma was isolated by centrifugation at 400 x g and 25 °C. All procedures were performed in accordance with the manufacturer´s protocol (Sigma Aldrich, 10771). Isolated plasma samples were subsequently stored at -80 °C, protected from light, until further analysis. Plasma isolation was conducted over a two-month period in 2022. A comprehensive overview of all patients and their respective diagnoses is provided in Supplementary Table 1.

### Sample preparation

Serum samples were processed to remove 14 of the most abundant plasma proteins using the High Select™ Depletion Spin Columns (P/N: A36370, ThermoFisher Scientific). This depletion step was performed according to the manufacturer’s protocol with 10 µl starting material.

Proteins collected in the flow-through were reduced by adding 40 µl of 100 mM dithiothreitol in ABC-buffer (ammonium-bicarbonate buffer, 100 mM, pH 8.0) and incubated at 56 °C for 30 min, followed by alkylation using 40 µl of 550 mM iodoacetamide in ABC-buffer, with the reaction proceeding at room temperature for 20 min in the dark. Proteins were then buffer exchanged using acetonitrile precipitation to 100 µl of 100 mM TEAB-buffer (triethylammonium-bicarbonate buffer 100 mM, pH8.5). The acetonitrile precipitation was performed as described elsewhere [38]. Samples were digested with 2 µg of trypsin (Sequencing Grade Modified Trypsin, P/N: V5111, Promega) for 6 h at 37 °C under constant agitation.

Peptides were labeled using the TMTpro™ 16plex Label Reagent Set (P/N A44521, ThermoFisher Scientific) following the manufacturer’s guidelines. After labelling, samples belonging to the same TMT multiplex were combined, lyophilized, and reconstituted in 60 µl of 0.1% formic acid.

Peptides were fractionated under high-pH conditions via reversed-phase chromatography using a XBridge Peptide BEH C18 column, 4.6 mm x 250 mm, 300 Å, 5 μm (P/N 186003625, Waters) as described previously [39].

A total of 47 fractions were initially collected, which were then concatenated into 16 pools. These pooled fractions were lyophilized and stored at -20 °C until further use. Prior to nanoLC-MS analysis, the peptides were re-dissolved in 0.1% formic acid, with 5% of each pool injected into the system.

### NanoLC-MS analysis

The peptide digests were analyzed on an UltiMate 3000 nano-HPLC system coupled to an Orbitrap Eclipse mass spectrometer (Thermo Scientific) equipped with a FAIMS (field asymmetric ion mobility spectrometer) interface as described previously [40]. The FAIMS device was operated at compensation voltages of -45, -55, and − 75 V, with each cycle lasting one second. Full-scan MS spectra were acquired at a resolution of 60,000 with an AGC target of 400,000. MS2 spectra were acquired in the Orbitrap at a resolution of 50,000, using an AGC target of 100,000 and an isolation window of m/z 0.7. Fragmentation was carried out via higher-energy collisional dissociation (HCD) using a normalized collision energy of 38.

### Database search

The MS data files were processed with Proteome Discoverer version 3.1 (Thermo Scientific). MS/MS spectra were searched using the Sequest HT algorithm against the Uniprot human reference proteome database (last modified 27/03/2024). The search settings were as follows: Enzyme specificity was set to trypsin, allowing up to two missed cleavages. Fixed modification was carbamidomethyl on cysteine; variable modifications were TMT labeled lysine and peptide N-terminus, oxidation of methionine, and acetylation and/or loss of the initiating methionine at the protein N-terminus. Mass tolerance was set to 10 ppm for precursor ions and 20 mmu for fragment ions. The maximum false discovery rate (FDR) for protein and peptide identification was set to 1%. For quantification, protein fold changes were determined from the intensity values of the 16 TMTpro reporter ions extracted from the MS2 spectra, using standard software settings. Protein abundances were normalized to total protein amount. No batch correction was applied, and missing values were excluded from the analysis. Maximum peptide co-isolation interference was set to 20%. Statistical significance of TMT reporter ion ratios was calculated in Proteome Discoverer using a t-test based on biological replicates. Resulting p-values were adjusted for multiple hypothesis testing using the Benjamini–Hochberg procedure.

The proteome analysis was done with Venny 2.1 (Oliveros, J.C. Venny; interactive tool for comparing lists with Venn´s diagram; https://bioinfogp.cnb.csic.es/tools/venny/index.html). We generated protein-protein interaction networks and clusters using the STRING database (https://string-db.org/; version 12.0). The analysis included proteins with expression changes greater than twofold, employing a permissive confidence threshold of *p* < 0.1 to avoid excluding potentially relevant proteins in the early stages of network analysis. A medium confidence threshold (0.4) was applied, and the strength of data support for the reported interactions is indicated by the thickness of the connecting lines.

### Subjects

Temporal artery biopsies (TABs) from three patients with PMR, three with extracranial GCA (EC-GCA), three with cranial GCA (C-GCA), and three controls (C) were provided by the Department of Pathology of University Hospital La Princesa (UHLP). At the time of TAB sampling, all clinical and laboratory data were recorded, and the final diagnosis was subsequently confirmed (Supplementary Table 4). PMR and GCA cases fulfill the respective classification criteria of the European Alliance of Associations for Rheumatology (EULAR 2022) [41] or the American College of Rheumatology (ACR 1990) [42]. Patients with EC-GCA were diagnosed with extra-cranial large-vessel vasculitis (LVV) using imaging modalities (CT-angiography, PET-CT, or ultrasound), while their TABs yielded negative results on routine haematoxylin and eosin staining (H&E). Patients with C-GCA had either a positive TAB or image evaluation. PMR patients exhibited no abnormalities in either TAB findings or imaging studies and did not present with cranial or extra-cranial ischemic manifestations. Furthermore, no clinical features suggestive of GCA developed in the PMR group during a one-year follow-up period. Active disease or flares in our patients were defined by characteristic clinical manifestations, regardless of whether inflammatory markers were elevated at the time of sampling. Conversely, patients with inactive disease presented with both normal clinical and laboratory findings. Control participants, who received an alternative diagnosis following biopsy, were selected to match the patient cohorts by age.

### Specimens´ preparation and immunofluorescence of temporal arteries

TAB specimens were sectioned at a thickness of 4 μm, mounted on glass slides, and preserved under a protective paraffin layer. In C-GCA patients, prior H&E staining revealed characteristic histopathological features, including intimal hyperplasia with luminal narrowing, fragmentation of the internal elastic membrane (IEM), and infiltration of macrophages and sporadically multinucleated giant cells (MNGCs).

For immunofluorescence analysis, sections were deparaffinized and subjected to heat-induced epitope retrieval by incubation at 96 °C for 14 min. Slides were subsequently washed in TBS-T (50 nm Tris-buffered saline, pH 7.2, 0.4% Triton X-100) and blocked for one hour with TBS-T containing 5% normal goat serum (NGS). Primary antibodies (S100A12, Sigma Aldrich, ZRB2595, and CD68, Sigma Aldrich, AMAb90874) were diluted 1:200 in TBS-T/5% NGS and applied for 20 h at 4 °C. Following washing, sections were incubated for two hours at room temperature with secondary antibodies (Alexa Fluor™ 488, Invitrogen, A-11034 and Alexa Fluor™ 594, Invitrogen, A-11032) diluted 1:1000 in TBS-T/5% NGS. After additional washing steps, slides were mounted using Fluorshield™ (Sigma Aldrich, F6182).

Images were acquired using a ReconCellVivo fluorescence microscope equipped with the CellSens Dimension software. Antigen expression was quantified using ImageJ (FijiWin64/ImageJ2015). Cells expressing S100A12 within the tunica adventitia or tunica media were manually counted, and mean values with standard deviations were calculated.

## Supplementary Information

Supplementary Material 1: Supplementary Table 1: Clinical features of patients included in the MS study. Supplementary Table 2: Significantly up- and downregulated plasma proteins identified in patients with active PMR based on volcano plot analysis. Supplementary Table 3: Significantly up- and downregulated plasma proteins identified in patients with active GCA based on volcano plot analysis. Supplementary Table 4: Clinical features of patients included in the immunofluorescence study.

## Data Availability

All data supporting the findings of this study are available within the paper and its Supplementary Information.

## Abbreviations

CRP: C-reactive protein
ESR: Erythrocyte sedimentation rate
GCA: Giant cell arteritis
GC: glucocorticoids
PBMCs: peripheral blood mononuclear cells
PMR: Polymyalgia rheumatica
TAB: temporal artery biopsy
