# Supplementary material for: Plasma proteome differences between giant cell arteritis and polymyalgia rheumatica: a pilot study
Source: Arthritis Res Ther. 2026 Jul 1;28:173. doi: 10.1186/s13075-026-03852-4 (PMC13587367; doi:10.1186/s13075-026-03852-4)
Supplement: Supplementary file 1 [file 13075_2026_3852_MOESM1_ESM.pdf]

Supplementary Table 1. List of clinical features and medications in the cohort of the MS study.

MDD	Age	Sex	Class.	Diagnosis	Clinical symptoms to diagnose the disease	Medication
60	78	F	Inactive	PMR	Polyarthralgia and asthenia	Prednisone 15mg/d
77	87	F	Inactive	PMR	Weakness in shoulder girdle and pelvic girdle	Prednisone 15 mg/d
96	68	F	Inactive	PMR	Pain in the neck and pelvic girdle	Prednisone 15 mg/d
52	60	F	Inactive	PMR	Pain in the neck, shoulder girdle, pelvis, and thighs	LEF, Prednisone 20mg/d
120	84	F	Inactive	PMR	Weakness in the shoulder girdle and pelvis	Prednisone 20 mg/d
1	77	F	Active	PMR	Arthralgia in the hands, neck pain, and pain in the shoulder girdle	Prednisone 10mg/d
1	85	M	Active	PMR	Pain in the shoulder girdle	Prednisone 10 mg/d
1	79	M	Active	PMR	Neck pain, weakness in the shoulder girdle, and pelvis	Prednisone 20 mg/d
1	75	F	Active	PMR	Neck pain, shoulder girdle pain, and pelvic pain	Prednisone 7.5mg/d
1	70	F	Active	PMR	Pain in the pelvic girdle and weakness in the lower extremities	Prednisone 30mg/d
7	79	F	Active	PMR	Pain in both girdles	MTX, Prednisone 7.5 mg/d
3	77	F	Active	PMR	Weakness in both girdles	Prednisone 10 mg/d
1	83	M	Active	PMR	Neck pain, shoulder girdle pain, and pelvic pain	Prednisone 25mg/d
1	75	M	Active	PMR	Neck pain, shoulder girdle pain, and pelvic pain	Prednisone 22mg/d
1	75	M	Active	PMR	Neck pain, shoulder girdle pain, and pelvic pain	Prednisone 8mg/d
135	87	F	Inactive	GCA	Vision loss	MTX, Prednisone 45 mg/d
84	82	M	Inactive	GCA	Frontotemporal headache, dysesthesia in the temporal region	MTX, Prednisone 60mg/d
84	90	F	Inactive	GCA	Headache	MTX, TCZ, Prednisone 50mg/d
41	91	F	Inactive	GCA	Left hemicranial headache, weakness in both waist areas	TCZ, Prednisone 40mg/d
27	67	M	Inactive	GCA	Pain and stiffness in both waist areas	MTX, TCZ, Deflazacort 30mg/d
150	77	F	Inactive	GCA	Headache, pain in waist areas	MTX, AZA, LEF, ETN, CYC, TCZ, Dacortin 40mg/d
1	78	M	Active	GCA	Headache	Prednisone 60mg/d
3	89	F	Active	GCA	Vision loss	Prednisone 30mg/d
122	92	F	Active	GCA	Pain in both waist areas	AZA, Prednisone 30mg/d
54	81	F	Active	GCA	Headache	TCZ, Prednisone 30mg/d
2	74	M	Active	GCA	Weakness in waist areas, asthenia	Prednisone 50mg/d
1	90	M	Active	GCA	Headache	TCZ, Prednisone 50mg/d
1	86	F	Active	GCA	Vision loss	Urbasone 80mg/d
-	80	F	-	C		-
-	79	F	-	C		-
-	78	F	-	C		-
-	75	F	-	C		-

C: control; PMR: polymyalgia rheumatica; GCA: giant cell arteritis; MDD: Mean disease duration (month); Class: classification active/inactive; F: female; M: male; MTX: methotrexate; TCZ: tocilizumab; AZA: azathioprine; CYC: cyclophosphamide; LEF: leflunomide; ETN: etanercept.

Supplementary Table 2. List of plasma proteins in active PMR that were downregulated (log_2 FC: -1.83 to -0.50) or upregulated (log_2 FC: 0.50 to 3.58) in the volcano plot analysis. abbr: abbreviation; act: active; con: control; ratio: proportion of the active group relative to the control; t-test: statistical comparison of group means; FC: fold change; normalized value threshold 0.05 - 0.95; t-test threshold: 0.1.

abbr.	description	ratio act/con	t-test act/con p-value	log2 FC	-log10 p-value	Benjamini-Hochberg p-value
SFN	14-3-3 protein sigma	0.282	0.0083	-1.8262	2.0792	0,0108
MINDY4B	Inactive ubiquitin carboxyl-terminal hydrolase MINDY-4B	0.285	0.0371	-1.8109	1.4308	0,0030
SLC4A1	Band 3 anion transport protein	0.342	0.0018	-1.5479	2.7342	0,0011
CAPZA2	F-actin-capping protein subunit alpha-2	0.344	0.0331	-1.5395	1.4801	0,0005
EVPL	Envoplakin	0.356	0.0257	-1.4900	1.5898	0,0005
SPTA1	Spectrin alpha chain. erythrocytic 1	0.375	0.0017	-1.4150	2.7584	0,0048
SCGB3A2	Secretoglobin family 3A member 2	0.41	0.0631	-1.2863	1.1996	0,0387
ANK1	Ankyrin-1	0.442	0.0014	-1.1778	2.8406	0,0054
SPTB	Spectrin beta chain. erythrocytic	0.464	0.0021	-1.1078	2.6707	0,0297
CYP27C1	Cytochrome P450 27C1	0.471	0.0145	-1.0862	1.8388	0,0862
FUCA2	Plasma alpha-L-fucosidase	0.498	0.0116	-1.0057	1.9359	0,0853
SLC2A1	Solute carrier family 2. facilitated glucose transporter member 1	0.51	0.0155	-0.9714	1.8096	0,1843
PLEKHH2	Pleckstrin homology domain-containing family H member 2	0.515	0.0265	-0.9573	1.5769	0,2724
MADCAM1	Mucosal addressin cell adhesion molecule 1	0.524	0.0008	-0.9323	3.1131	0,3563
H2AX	Histone H2AX	0.529	0.0544	-0.9186	1.2641	0,3567
LOXL3	Lysyl oxidase homolog 3	0.536	0.0408	-0.8996	1.3898	0,4491
EEF1A1	Elongation factor 1-alpha 1	0.546	0.0850	-0.8730	1.0703	0,3125
PGM2	Phosphopentomutase	0.551	0.0694	-0.8598	1.1584	0,1638
HAL	Histidine ammonia-lyase	0.555	0.0837	-0.8494	1.0773	0,1938
PCYOX1	Prenylcysteine oxidase 1	0.557	0.0016	-0.8442	2.8058	0,3122
MED30	Mediator of RNA polymerase II transcription subunit 30	0.557	0.0093	-0.8442	2.0326	0,1769
KRT14	Keratin. type I cytoskeletal 14	0.567	0.0887	-0.8185	1.0521	0,4249
KRT10	Keratin. type I cytoskeletal 10	0.574	0.0384	-0.8008	1.4155	0,4724
LGALS7	Galectin-7	0.576	0.0008	-0.7958	3.1235	0,2300
SHBG	Sex hormone-binding globulin	0.579	0.0062	-0.7883	2.2074	0,2379
CADPS2	Calcium-dependent secretion activator 2	0.579	0.0243	-0.7883	1.6146	0,6019
PRKAA1	5'-AMP-activated protein kinase catalytic subunit alpha-1	0.582	0.0213	-0.7809	1.6719	0,1791
RHCE	Blood group Rh(CE) polypeptide	0.586	0.0073	-0.7710	2.1345	0,4166
CBLN1	Cerebellin-1	0.593	0.0162	-0.7538	1.7894	0,4806
HEBP1	Heme-binding protein 1	0.6	0.0203	-0.7369	1.6932	0,2065
CA1	Carbonic anhydrase 1	0.6	0.0859	-0.7369	1.0659	0,4164
PRXL2A	Peroxiredoxin-like 2A	0.605	0.0335	-0.7249	1.4747	0,2527
EPB42	Protein 4.2	0.609	0.0047	-0.7154	2.3289	0,2065
PLBD1	Phospholipase B-like 1	0.618	0.0586	-0.6943	1.2323	0,3541
UMOD	Uromodulin	0.624	0.0958	-0.6803	1.0185	0,2706
ANTXR1	Anthrax toxin receptor 1	0.627	0.0082	-0.6734	2.0880	0,7263
EXTL2	Exostosin-like 2	0.627	0.0192	-0.6734	1.7168	0,3350
GXYLT1	Glucoside xylosyltransferase 1	0.632	0.0395	-0.6620	1.4036	0,3788
HRG	Histidine-rich glycoprotein	0.632	0.0532	-0.6620	1.2743	0,3535
LCN1	Lipocalin-1	0.632	0.0906	-0.6620	1.0429	0,7270
PON1	Serum paraoxonase/arylesterase 1	0.634	0.0411	-0.6574	1.3859	0,4866
PLD4	5'-3' exonuclease PLD4	0.639	0.0261	-0.6461	1.5828	0,4983
COL11A2	Collagen alpha-2(XI) chain	0.64	0.0602	-0.6438	1.2205	0,3828
EPB41	Protein 4.1	0.643	0.0351	-0.6371	1.4543	0,0923
TFAP2D	Transcription factor AP-2-delta	0.647	0.0782	-0.6281	1.1070	0,5085
FBXO7	F-box only protein 7	0.649	0.0722	-0.6237	1.1412	0,2489
RAB3C	Ras-related protein Rab-3C	0.651	0.0533	-0.6192	1.2733	0,5841
FUCA1	Tissue alpha-L-fucosidase	0.655	0.0413	-0.6104	1.3843	0,3770
MUCL1	Mucin-like protein 1	0.656	0.0955	-0.6082	1.0200	0,5571
IL1RAP	Interleukin-1 receptor accessory protein	0.659	0.0349	-0.6016	1.4573	0,5825
KIT	Mast/stem cell growth factor receptor Kit	0.661	0.0011	-0.5972	2.9508	0,5589
ARSG	Arylsulfatase G	0.668	0.0719	-0.5820	1.1433	0,5899
CNDP1	Beta-Ala-His dipeptidase	0.671	0.0078	-0.5756	2.1069	0,6173
HSD11B1	11-beta-hydroxysteroid dehydrogenase 1	0.674	0.0644	-0.5691	1.1913	0,6023
SELENBP1	Methanethiol oxidase	0.676	0.0266	-0.5649	1.5748	0,3770
APOB	Apolipoprotein B-100	0.676	0.0513	-0.5649	1.2895	0,5502
CNTN1	Contactin-1	0.683	0.0010	-0.5500	3.0159	0,5722
PON3	Serum paraoxonase/lactonase 3	0.684	0.0028	-0.5479	2.5558	0,4954
C1QTNF3	Complement C1q tumor necrosis factor-related protein 3	0.684	0.0288	-0.5479	1.5402	0,5469
ITGA7	Integrin alpha-7	0.689	0.0177	-0.5374	1.7515	0,5645
CETP	Cholesteryl ester transfer protein	0.69	0.0357	-0.5353	1.4472	0,6398
PRDX2	Peroxiredoxin-2	0.698	0.0823	-0.5187	1.0846	0,3488
PLA2G7	Platelet-activating factor acetylhydrolase	0.702	0.0663	-0.5104	1.1782	0,6023
FLG2	Filaggrin-2	0.706	0.0177	-0.5022	1.7516	0,6405
DEFA6	Defensin-6	1.415	0.0516	0.5008	1.2876	0,7305
IGFBP6	Insulin-like growth factor-binding protein 6	1.415	0.0590	0.5008	1.2291	0,7483
YIPF3	Protein YIPF3	1.42	0.0782	0.5058	1.1065	0,7909
CALR	Calreticulin	1.426	0.0093	0.5119	2.0311	0,6735
PYGM	Glycogen phosphorylase. muscle form	1.427	0.0408	0.5129	1.3893	0,7810
HLA-B	HLA class I histocompatibility antigen. B alpha chain	1.43	0.0443	0.5160	1.3533	0,4249

AZGP1	Zinc-alpha-2-glycoprotein	1.433	0.0494	0.5190	1.3063	0,8136
SH3BGRL	Adapter SH3BGRL	1.439	0.0280	0.5250	1.5530	0.7293
VBP1	Prefoldin subunit 3	1.449	0.0725	0.5350	1.1397	0,4677
ATF6B	Cyclic AMP-dependent transcription factor ATF-6 beta	1.45	0.0415	0.5360	1.3821	0,7470
CFHR5	Complement factor H-related protein 5	1.457	0.0686	0.5430	1.1639	0,5454
TNFRSF21	Tumor necrosis factor receptor superfamily member 21	1.458	0.0655	0.5439	1.1839	0,6233
CSF1	Macrophage colony-stimulating factor 1	1.46	0.0627	0.5459	1.2024	0,6585
ANXA5	Annexin A5	1.477	0.0697	0.5626	1.1570	0,8136
LSP1	Lymphocyte-specific protein 1	1.483	0.0028	0.5685	2.5591	0,3504
PTRHD1	Putative peptidyl-tRNA hydrolase PTRHD1	1.515	0.0532	0.5993	1.2743	0,5910
MARCKS	Myristoylated alanine-rich C-kinase substrate	1.516	0.0111	0.6002	1.9565	0,8202
MYDGF	Myeloid-derived growth factor	1.52	0.0591	0.6040	1.2284	0,7104
HLA-A	HLA class I histocompatibility antigen. A alpha chain	1.524	0.0387	0.6078	1.4127	0,4008
CNBP	CCHC-type zinc finger nucleic acid binding protein	1.534	0.0535	0.6172	1.2719	0,9481
HSPA1B	Heat shock 70 kDa protein 1B	1.549	0.0898	0.6313	1.0465	0,5763
CORO1A	Coronin-1A	1.556	0.0398	0.6378	1.4006	0,5698
SCFD1	Sec1 family domain-containing protein 1	1.556	0.0871	0.6378	1.0598	0,1814
VSIG4	V-set and immunoglobulin domain-containing protein 4	1.557	0.0732	0.6387	1.1354	0,7299
UGGT1	UDP-glucose:glycoprotein glucosyltransferase 1	1.561	0.0218	0.6424	1.6613	0,8709
EWSR1	RNA-binding protein EWS	1.561	0.0916	0.6424	1.0381	0,4947
IGHV3-74	Immunoglobulin heavy variable 3-74	1.564	0.0917	0.6452	1.0374	0,3958
PRAP1	Proline-rich acidic protein 1	1.565	0.0124	0.6461	1.9078	0,7299
PCSK1N	ProSAAS	1.58	0.0438	0.6599	1.3585	0,8437
ANGPTL4	Angiopoietin-related protein 4	1.581	0.0535	0.6608	1.2715	0,5821
S100P	Protein S100-P	1.582	0.0166	0.6617	1.7800	0,6518
GUCA2A	Guanylin	1.583	0.0391	0.6626	1.4079	0,3682
FCRL5	Fc receptor-like protein 5	1.584	0.0158	0.6635	1.8012	0,2737
REG3A	Regenerating islet-derived protein 3-alpha	1.588	0.0882	0.6672	1.0546	0,7907
UBA1	Ubiquitin-like modifier-activating enzyme 1	1.592	0.0519	0.6708	1.2846	0,6264
LOX	Protein-lysine 6-oxidase	1.6	0.0411	0.6780	1.3859	0,4954
	Immunoglobulin kappa light chain	1.601	0.0667	0.6789	1.1760	0,4736
SERPINA1	Alpha-1-antitrypsin	1.615	0.0100	0.6915	2.0018	0,4201
IGHV3-35	Probable non-functional immunoglobulin heavy variable 3-35	1.617	0.0816	0.6933	1.0884	0,5040
RANBP1	Ran-specific GTPase-activating protein	1.62	0.0011	0.6959	2.9554	0,1728
HPR	Haptoglobin-related protein	1.62	0.0339	0.6959	1.4703	0,3788
TMED10	Transmembrane emp24 domain-containing protein 10	1.639	0.0542	0.7128	1.2658	0,3573
GNAI2	Guanine nucleotide-binding protein G(i) subunit alpha-2	1.641	0.0166	0.7145	1.7804	0,2533
EXOC7	Exocyst complex component 7	1.673	0.0664	0..4243	1.1775	0,3366
ARPC4	Actin-related protein 2/3 complex subunit 4	1.675	0.0994	0.7441	1.0028	0,4044
TBCA	Tubulin-specific chaperone A	1.692	0.0200	0.7587	1.6994	0,1931
DNAJC10	DnaJ homolog subfamily C member 10	1.695	0.0133	0.7612	1.8773	0,2341
CCT6A	T-complex protein 1 subunit zeta	1.7	0.0021	0.6553	2.6762	0,2904
NUCB2	Nucleobindin-2	1.7	0.0213	0.7655	1.6711	0,3763
YWHAH	14-3-3 protein eta	1.701	0.0282	0.7663	1.5501	0,5410
ACTR2	Actin-related protein 2	1.707	0.0373	0.7714	1.4282	0,5492
GPI	Glucose-6-phosphate isomerase	1.71	0.0104	0.7739	1.9813	0,4640
GGT1	Glutathione hydrolase 1 proenzyme	1.714	0.0074	0.7773	2.1331	0,3788
VCL	Vinculin	1.735	0.0879	0.7949	1.0559	0,1304
TWF2	Twinfilin-2	1.737	0.0538	0.7965	1.2688	0,2213
TUBA3C	Tubulin alpha-3C chain	1.738	0.0264	0.7974	1.5784	0,4201
SLPI	Antileukoproteinase	1.756	0.0263	0.8122	1.5804	0,2486
PARVB	Beta-parvin	1.757	0.0946	0.8131	1.0240	0,2330
DEFA1	Neutrophil defensin 1	1.764	0.0023	0.8188	2.6366	0,2173
CFHR3	Complement factor H-related protein 3	1.769	0.0590	0.8229	1.2294	0,4046
MANSC1	MANSC domain-containing protein 1	1.773	0.0446	0.8261	1.3506	0,5164
DCTN1	Dynactin subunit 1	1.775	0.0299	0.8278	1.5240	0,1229
NFKB1	Nuclear factor NF-kappa-B p105 subunit	1.784	0.0240	0.8351	1.6196	0,4962
SLC39A10	Zinc transporter ZIP10	1.792	0.0544	0.8415	1.2647	0,5083
PSMB2	Proteasome subunit beta type-2	1.802	0.0341	0.8495	1.4670	0,4492
LTBP3	Latent-transforming growth factor beta-binding protein 3	1.804	0.0631	0.8511	1.1997	0,2065
LMNB1	Lamin-B1	1.808	0.0136	0.8543	1.8665	0,3994
CGREF1	Cell growth regulator with EF hand domain protein 1	1.829	0.0944	0.8710	1.0250	0,3042
ARPC1B	Actin-related protein 2/3 complex subunit 1B	1.848	0.0802	0.8859	1.0958	0,1627
HSPE1	10 kDa heat shock protein. mitochondrial	1.853	0.0172	0.8898	1.7636	0,2640
RNASE2	Non-secretory ribonuclease	1.855	0.0261	0.8914	1.5828	0,1727
ACTC1	Actin. alpha cardiac muscle 1	1.858	0.0779	0.8937	1.1084	0,1747
TAF15	TATA-binding protein-associated factor 2N	1.88	0.0755	0.9107	1.1217	0,1728
EIF2B1	Translation initiation factor eIF2B subunit alpha	1.889	0.0471	0.9176	1.3273	0,1927
IGKV3D-20	Immunoglobulin kappa variable 3D-20	1.899	0.0532	0.9252	1.2741	0,1219
UBE2L3	Ubiquitin-conjugating enzyme E2 L3	1.9	0.0010	0.9259	2.9831	0,1120
GRB2	Growth factor receptor-bound protein 2	1.908	0.0092	0.9320	2.0339	0,1767
ACTR3	Actin-related protein 3	1.916	0.0641	0.9380	1.1928	0,0328
NRGN	Neurogranin	1.922	0.0777	0.9426	1.1093	0,2125
SERPINA3	Alpha-1-antichymotrypsin	1.937	0.0100	0.9538	2.0009	0,2426
SDF4	45 kDa calcium-binding protein	1.952	0.0160	0.9649	1.7946	0,0931
NAP1L4	Nucleosome assembly protein 1-like 4	1.975	0.0031	0.9818	2.5128	0,1791
CANX	Calnexin	1.975	0.0281	0.9818	1.5519	0,1843
WDR1	WD repeat-containing protein 1	1.977	0.0833	0.9833	1.0792	0,0976
GP6	Platelet glycoprotein VI	1.985	0.0005	0.9891	3.2758	0,0818
HP	Haptoglobin	1.985	0.0384	0.9891	1.4161	0,1785
ARPC5	Actin-related protein 2/3 complex subunit 5	1.991	0.0266	0.9934	1.5749	0,0364
ARF3	ADP-ribosylation factor 3	2.003	0.0001	1.0021	3.9150	0,2486
CLIC1	Chloride intracellular channel protein 1	2.01	0.0439	1.0071	1.3574	0,1424

MYLK	Myosin light chain kinase. smooth muscle	2.025	0.0182	1.0179	1.7401	0,4677
COTL1	Coactosin-like protein	2.025	0.0309	1.1792	1.5095	0.3054
MMP3	Stromelysin-1	2.025	0.0316	1.0179	1.5006	0,1889
ZYX	Zyxin	2.033	0.0727	1.0236	1.1383	0,1424
COLEC12	Collectin-12	2.04	0.0321	1.0285	1.4930	0,1068
PFN1	Profilin-1	2.042	0.0559	1.0299	1.2527	0,2895
VIM	Vimentin	2.046	0.0627	1.0328	1.2030	0,2873
TMSB4X	Thymosin beta-4	2.068	0.0288	1.0482	1.5403	0,0826
TMX1	Thioredoxin-related transmembrane protein 1	2.104	0.0026	1.0731	2.5847	0,0225
CKAP5	Cytoskeleton-associated protein 5	2.104	0.0576	1.0731	1.2394	0,0084
PI3	Elafin	2.109	0.0503	1.0765	1.2981	0,2046
CCT8	T-complex protein 1 subunit theta	2.139	0.0005	1.0969	3.2941	0,0374
TAGLN2	Transgelin-2	2.142	0.0561	1.0989	1.2508	0,0519
EFHD2	EF-hand domain-containing protein D2	2.147	0.0095	1.1023	2.0242	0,0505
RAB18	Ras-related protein Rab-18	2.158	0.0112	1.1096	1.9525	0,0103
TPM1	Tropomyosin alpha-1 chain	2.163	0.0984	1.1130	1.0070	0,0740
RPN1	Dolichyl-diphosphooligosaccharide--protein glycosyltransferase subunit 1	2.181	0.0088	1.1249	2.0576	0,0172
SERPINB1	Leukocyte elastase inhibitor	2.193	0.0125	1.1329	1.9022	0,2213
MT1G	Metallothionein-1G	2.199	0.0128	1.1368	1.8944	0,1914
KPNB1	Importin subunit beta-1	2.213	0.0212	1.1460	1.6737	0,0826
SH3BGRL3	SH3 domain-binding glutamic acid-rich-like protein 3	2.264	0.0128	1.1788	1.8913	0,1281
ARHGDIB	Rho GDP-dissociation inhibitor 2	2.282	0.0077	1.1902	2.1113	0,1545
CAPZB	F-actin-capping protein subunit beta	2.283	0.0086	1.1909	2.0659	0,0389
CHI3L1	Chitinase-3-like protein 1	2.318	0.0694	1.2128	1.1584	0,2100
MAPRE1	Microtubule-associated protein RP/EB family member 1	2.344	0.0547	1.2289	1.2623	0,1304
LEAP2	Liver-expressed antimicrobial peptide 2	2.375	0.0017	1.2479	2.7687	0,1168
CAVIN2	Caveolae-associated protein 2	2.375	0.0683	1.2479	1.1652	0,0369
S100A9	Protein S100-A9	2.387	0.0046	1.2551	2.3365	0,0255
CAPZA1	F-actin-capping protein subunit alpha-1	2.404	0.0059	1.2654	2.2258	0,0092
LASP1	LIM and SH3 domain protein 1	2.42	0.0205	1.2750	1.6883	0,1017
TPT1	Translationally-controlled tumor protein	2.423	0.0036	1.2767	2.4473	0,0897
GP1BB	Platelet glycoprotein Ib beta chain	2.43	0.0811	1.2809	1.0907	0,0776
ORM2	Alpha-1-acid glycoprotein 2	2.485	0.0013	1.3132	2.8790	0,0129
ANXA3	Annexin A3	2.485	0.0020	1.3132	2.6994	0,0282
CASP3	Caspase-3	2.486	0.0214	1.3138	1.6699	0,0350
HSPD1	60 kDa heat shock protein. mitochondrial	2.497	0.0015	1.3201	2.8366	0,0021
VASP	Vasodilator-stimulated phosphoprotein	2.515	0.0701	1.3305	1.1542	0,0196
PPP6C	Serine/threonine-protein phosphatase 6 catalytic subunit	2.582	0.0143	1.3684	1.8457	0,0088
MT-CO2	Cytochrome c oxidase subunit 2	2.596	0.0008	1.3762	3.1053	0,0003
PRTFDC1	Phosphoribosyltransferase domain-containing protein 1	2.612	0.0155	1.3851	1.8094	0,0092
PTGES3	Prostaglandin E synthase 3	2.646	0.0002	1.4038	3.6994	0,0037
TXNDC12	Thioredoxin domain-containing protein 12	2.655	0.0082	1.4087	2.0847	0,0021
PDLIM1	PDZ and LIM domain protein 1	2.682	0.0733	1.4233	1.1347	0,0063
ORM1	Alpha-1-acid glycoprotein 1	2.724	0.0030	1.4457	2.5253	0,0007
MYL6B	Myosin light chain 6B	2.756	0.0702	1.4625	1.1537	0,0206
DBN1	Drebrin	2.798	0.0221	1.4843	1.6560	0,0091
LRRC59	Leucine-rich repeat-containing protein 59	2.887	0.0015	1.5295	2.8102	0,0003
TUBA8	Tubulin alpha-8 chain	2.899	0.0055	1.5355	2.2622	0,0000
MTPN	Myotrophin	2.936	0.0021	1.5538	2.6831	0,0007
S100A8	Protein S100-A8	3.012	0.0065	1.5907	2.1871	0,0040
SNAP23	Synaptosomal-associated protein 23	3.024	0.0005	1.5964	3.3453	0,0001
TPM3	Tropomyosin alpha-3 chain	3.097	0.0182	1.6308	1.7388	0,0005
AP1G1	AP-1 complex subunit gamma-1	3.13	0.0022	1.6461	2.6560	0,0002
MYL12B	Myosin regulatory light chain 12B	3.152	0.0988	1.6562	1.0053	0,0012
DBI	Acyl-CoA-binding protein	3.201	0.0024	1.6785	2.6197	0,0011
SAFB2	Scaffold attachment factor B2	3.289	0.0923	1.7176	1.0350	0,0005
BIN2	Bridging integrator 2	3.293	0.0202	1.7194	1.6937	0,0012
S100A12	Protein S100-A12	3.294	0.0043	1.7198	2.3712	0,0013
SAA2	Serum amyloid A-2 protein	3.518	0.0854	1.8147	1.0682	0,0000
MYADM	Myeloid-associated differentiation marker	3.546	0.0070	1.8261	2.1552	0,0000
ATP2A3	Sarcoplasmic/endoplasmic reticulum calcium ATPase 3	3.652	0.0090	1.8686	2.0481	0,0001
TPM4	Tropomyosin alpha-4 chain	3.767	0.0617	1.9134	1.2099	0,0000
TFF1	Trefoil factor 1	3.891	0.0050	1.9601	2.2972	0,0001
OSTF1	Osteoclast-stimulating factor 1	4.052	0.0226	2.0186	1.6464	0,0000
DDOST	Dolichyl-diphosphooligosaccharide--protein glycosyltransferase 48 kDa subunit	4.086	0.0022	2.0306	2.6537	0,0000
DBNL	Drebrin-like protein	4.567	0.0008	2.1912	3.0807	0,0000
ABCC8	ATP-binding cassette sub-family C member 8	5.06	0.0075	2.3391	2.1259	0,0000
RTN4	Reticulon-4	5.621	0.0009	2.4908	3.0302	0,0000
GP9	Platelet glycoprotein IX	5.66	0.0028	2.5008	2.5532	0,0000
PSTPIP2	Proline-serine-threonine phosphatase-interacting protein 2	5.718	0.0079	2.5155	2.1047	0,0000
RAB27B	Ras-related protein Rab-27B	6.249	0.0088	2.6436	2.0579	0,0000
CRP	C-reactive protein	6.537	0.0166	2.7086	1.7796	0,0000
SEPTIN2	Septin-2	6.699	0.0017	2.7439	2.7771	0,0000
IGHV2-70D	Immunoglobulin heavy variable 2-70D	8.473	0.0336	3.0828	1.4734	0,0000
SAA1	Serum amyloid A-1 protein	10.479	0.0428	3.3894	1.3690	0,0000
DNM1L	Dynamin-1-like protein	11.957	0.0133	3.5797	1.8771	0,0000

Supplementary Table 3. List of plasma proteins in active GCA that were downregulated (log_2 FC: -1.90 to -0.50) or upregulated (log_2 FC: 0.50 to 2.32) in the volcano plot analysis. abbr: abbreviation; act: active; con: control; ratio: proportion of the active group relative to the control; t-test: statistical comparison of group means; FC: fold change; normalized value threshold 0.05 - 0.95; t-test threshold: 0.1.

abbr.	description	ratio act/con	t-test act/con p-value	log2 FC	-log10 p-value	Benjamini -Hochberg p-value
FLT3	Receptor-type tyrosine-protein kinase FLT3	0.268	0.0070	-1.8996	2.1571	0,0011
XPNPEP2	Xaa-Pro aminopeptidase 2	0.377	0.0249	-1.4073	1.6036	0,0002
EIF2B1	Translation initiation factor eIF2B subunit alpha	0.382	0.0966	-1.3883	1.0148	0,0048
PROCR	Endothelial protein C receptor	0.384	0.0710	-1.3808	1.1485	0,1115
SCGB3A2	Secretoglobin family 3A member 2	0.402	0.0876	-1.3147	1.0575	0,0360
DCD	Dermcidin	0.426	0.0212	-1.2310	1.6735	0,2428
MUCL1	Mucin-like protein 1	0.434	0.0783	-1.2042	1.1062	0,0272
PLD4	5'-3' exonuclease PLD4	0.488	0.0370	-1.0350	1.4323	0,0495
IGF2	Insulin-like growth factor II	0.52	0.0777	-0.9434	1.1093	0,1189
ANTXR1	Anthrax toxin receptor 1	0.523	0.0519	-0.9351	1.2849	0,1215
LRP1B	Low-density lipoprotein receptor-related protein 1B	0.527	0.0584	-0.9241	1.2336	0,0577
CETP	Cholesteryl ester transfer protein	0.55	0.0285	-0.8624	1.5450	0,1038
KPRP	Keratinocyte proline-rich protein	0.553	0.0630	-0.8546	1.2007	0,3988
COL5A1	Collagen alpha-1(V) chain	0.568	0.0642	-0.8160	1.1926	0,1291
FASTKD5	FAST kinase domain-containing protein 5. mitochondrial	0.571	0.0839	-0.8084	1.0764	0,0629
HRG	Histidine-rich glycoprotein	0.603	0.0029	-0.7297	2.5328	0,2848
IGDCC4	Immunoglobulin superfamily DCC subclass member 4	0.604	0.0347	-0.7273	1.4597	0,2078
PI16	Peptidase inhibitor 16	0.61	0.0338	-0.7131	1.4704	0,0678
IL1RAP	Interleukin-1 receptor accessory protein	0.611	0.0037	-0.7107	2.4314	0,4053
KIT	Mast/stem cell growth factor receptor Kit	0.615	0.0044	-0.7013	2.3543	0,1111
CD209	CD209 antigen	0.618	0.0536	-0.6943	1.2708	0,5067
KIF13B	Kinesin-like protein KIF13B	0.621	0.0828	-0.6873	1.0817	0,3483
IGFBP3	Insulin-like growth factor-binding protein 3	0.623	0.0416	-0.6826	1.3812	0,0448
IGFALS	Insulin-like growth factor-binding protein complex acid labile subunit	0.632	0.0488	-0.6620	1.3117	0,1182
C1QTNF3	Complement C1q tumor necrosis factor-related protein 3	0.641	0.0494	-0.6416	1.3062	0,1206
CADPS2	Calcium-dependent secretion activator 2	0.645	0.0874	-0.6326	1.0585	0,4471
CPQ	Carboxypeptidase Q	0.647	0.0811	-0.6281	1.0910	0,2337
PON3	Serum paraoxonase/lactonase 3	0.654	0.0109	-0.6126	1.9615	0,2813
LRIG2	Leucine-rich repeats and immunoglobulin-like domains protein 2	0.66	0.0731	-0.5994	1.1361	0,3993
KRT77	Keratin. type II cytoskeletal 1b	0.662	0.0981	-0.5950	1.0083	0,5406
SHBG	Sex hormone-binding globulin	0.663	0.0989	-0.5929	1.0049	0,1110
HMCN2	Hemicentin-2	0.665	0.0807	-0.5885	1.0928	0,2008
GTF2IRD2	General transcription factor II-I repeat domain-containing protein 2A	0.671	0.0062	-0.5756	2.2081	0,1872
B4GAT1	Beta-1.4-glucuronyltransferase 1	0.673	0.0351	-0.5713	1.4543	0,4953
ADGRG2	Adhesion G-protein coupled receptor G2	0.681	0.0152	-0.5542	1.8184	0,5715
FLG2	Filaggrin-2	0.685	0.0649	-0.5458	1.1874	0,2413
FBXO7	F-box only protein 7	0.694	0.0049	-0.5269	2.3121	0,3993
ALB	Albumin	0.695	0.0685	-0.5249	1.1641	0,3416
GGH	Gamma-glutamyl hydrolase	0.698	0.0541	-0.5187	1.2670	0,4523
PCYOX1	Prenylcysteine oxidase 1	0.701	0.0044	-0.5125	2.3606	0,5928
LGALS1	Galectin-1	1.42	0.0597	0.5058	1.2239	0,7891
BSG	Basigin	1.421	0.0520	0.5069	1.2843	0,7703
PSMA1	Proteasome subunit alpha type-1	1.444	0.0834	0.5300	1.0787	0,6212
LTBP2	Latent-transforming growth factor beta-binding protein 2	1.446	0.0530	0.5320	1.2754	0,8459
ADGRF5	Adhesion G protein-coupled receptor F5	1.446	0.0662	0.5320	1.1792	0,6073
UBE2V1	Ubiquitin-conjugating enzyme E2 variant 1	1.458	0.0736	0.5439	1.1331	0,7182
SPINK1	Serine protease inhibitor Kazal-type 1	1.461	0.0190	0.5469	1.7212	0,1726
IGFBP6	Insulin-like growth factor-binding protein 6	1.461	0.0667	0.5469	1.1756	0,6869
GPT	Alanine aminotransferase 1	1.471	0.0784	0.5567	1.1059	0,8637
ANG	Angiogenin	1.473	0.0187	0.5587	1.7270	0,8211
CES2	Cocaine esterase	1.473	0.0787	0.5587	1.1039	0,8209
IGLV2-18	Immunoglobulin lambda variable 2-18	1.475	0.0858	0.5607	1.0663	0,8934
PECAM1	Platelet endothelial cell adhesion molecule	1.478	0.0665	0.5636	1.1772	0,0864
HPN	Serine protease hepsin	1.483	0.0591	0.5685	1.2281	0,7937
CCL15	C-C motif chemokine 15	1.489	0.0203	0.5743	1.6923	0,8132
NPPA	Natriuretic peptides A	1.493	0.0487	0.5782	1.3128	0,7611
NFKB1	Nuclear factor NF-kappa-B p105 subunit	1.502	0.0595	0.5868	1.2252	0,2484
NPTX2	Neuronal pentraxin-2	1.507	0.0541	0.5916	1.2668	0,7063
MPO	Myeloperoxidase	1.507	0.0826	0.5916	1.0831	0,5429
GPX1	Glutathione peroxidase 1	1.511	0.0297	0.5955	1.5270	0,4040
PIP4K2B	Phosphatidylinositol 5-phosphate 4-kinase type-2 beta	1.512	0.0874	0.5964	1.0585	0,3808
NUCB1	Nucleobindin-1	1.532	0.0205	0.6154	1.6878	0,5474
PGLS	6-phosphogluconolactonase	1.533	0.0002	0.6163	3.6572	0,8078
IGHA1	Immunoglobulin heavy constant alpha 1	1.533	0.0856	0.6163	1.0677	0,1273
TAGLN	Transgelin	1.535	0.0320	0.6182	1.4950	0,8308
ECE1	Endothelin-converting enzyme 1	1.546	0.0454	0.6285	1.3427	0,7019
EWSR1	RNA-binding protein EWS	1.546	0.0533	0.6285	1.2729	0,9416
PSMA7	Proteasome subunit alpha type-7	1.554	0.0887	0.6359	1.0519	0,6443
IL1R2	Interleukin-1 receptor type 2	1.556	0.0624	0.6378	1.2046	0,2484
CSTB	Cystatin-B	1.568	0.0556	0.6489	1.2550	0,3810
PSMB4	Proteasome subunit beta type-4	1.577	0.0105	0.6571	1.9802	0,5947
GNAS	Guanine nucleotide-binding protein G(s) subunit alpha isoforms XLas	1.593	0.0543	0.6717	1.2649	0,6643

ADA2	Adenosine deaminase 2	1.594	0.0228	0.6726	1.6413	0,5116
PYGM	Glycogen phosphorylase. muscle form	1.6	0.0011	0.6780	2.9487	0,4708
TBXAS1	Thromboxane-A synthase	1.612	0.0657	0.6888	1.1822	0,5715
ARHGDIA	Rho GDP-dissociation inhibitor 1	1.613	0.0792	0.6897	1.1013	0,6785
IL18	Interleukin-18	1.622	0.0385	0.6977	1.4148	0,5916
F8	Coagulation factor VIII	1.625	0.0032	0.7004	2.4929	0,3006
NHERF1	Na(+)/H(+) exchange regulatory cofactor NHE-RF1	1.64	0.0010	0.7136	2.9980	0,6402
FUOM	Fucose mutarotase	1.647	0.0874	0.7198	1.0585	0,4857
MYLK	Myosin light chain kinase. smooth muscle	1.653	0.0135	0.7250	1.8686	0,0987
ITM2B	Integral membrane protein 2B	1.656	0.0544	0.7277	1.2644	0,8934
SERPINA9	Serpin A9	1.66	0.0323	0.7311	1.4901	0,6869
IGLC3	Immunoglobulin lambda constant 3	1.661	0.0126	0.7320	1.9009	0,5720
PSMB8	Proteasome subunit beta type-8	1.666	0.0108	0.7363	1.9683	0,4798
LSP1	Lymphocyte-specific protein 1	1.668	0.0351	0.7381	1.4544	0,3218
GP6	Platelet glycoprotein VI	1.671	0.0099	0.7407	2.0062	0,4073
IFI35	Interferon-induced 35 kDa protein	1.679	0.0132	0.7476	1.8803	0,5177
GP2	Pancreatic secretory granule membrane major glycoprotein GP2	1.684	0.0163	0.7518	1.7865	0,4894
PSMD13	26S proteasome non-ATPase regulatory subunit 13	1.7	0.0510	0.7655	1.2926	0,6461
GPX3	Glutathione peroxidase 3	1.749	0.0550	0.8065	1.2593	0,2724
IGHV1-69	Immunoglobulin heavy variable 1-69	1.752	0.0846	0.8090	1.0727	0,4667
APOC3	Apolipoprotein C-III	1.761	0.0091	0.8163	2.0413	0,5219
PRAP1	Proline-rich acidic protein 1	1.771	0.0258	0.8245	1.5875	0,2188
VSIG4	V-set and immunoglobulin domain-containing protein 4	1.785	0.0684	0.8359	1.1651	0,4799
CORO1A	Coronin-1A	1.787	0.0442	0.8375	1.3541	0,0764
VIM	Vimentin	1.787	0.0611	0.8375	1.2139	0,7395
ACY1	Aminoacylase-1	1.807	0.0204	0.8535	1.6907	0,6674
PGK1	Phosphoglycerate kinase 1	1.82	0.0990	0.8639	1.0042	0,2867
GPI	Glucose-6-phosphate isomerase	1.828	0.0044	0.8702	2.3575	0,2091
NIBAN1	Protein Niban 1	1.839	0.0127	0.8789	1.8965	0,5038
SERPINA3	Alpha-1-antichymotrypsin	1.874	0.0295	0.9061	1.5298	0,0526
CEP290	Centrosomal protein of 290 kDa	1.888	0.0154	0.9168	1.8115	0,0602
CDC42	Cell division control protein 42 homolog	1.893	0.0088	0.9206	2.0549	0,0923
UBA1	Ubiquitin-like modifier-activating enzyme 1	1.904	0.0455	0.9290	1.3421	0,4036
G6PD	Glucose-6-phosphate 1-dehydrogenase	1.917	0.0487	0.9388	1.3127	0,3012
ANXA5	Annexin A5	1.926	0.0743	0.9456	1.1288	0,4225
ANXA3	Annexin A3	1.93	0.0019	0.9486	2.7238	0,0953
ENO1	Alpha-enolase	1.94	0.0589	0.9560	1.2299	0,0778
PTBP1	Polypyrimidine tract-binding protein 1	1.946	0.0291	0.9605	1.5354	0,6924
VWF	von Willebrand factor	1.956	0.0498	0.9679	1.3029	0,0246
YWHAG	14-3-3 protein gamma	1.995	0.0466	0.9963	1.3316	0,0925
ADM	Pro-adrenomedullin	2.002	0.0229	1.0014	1.6396	0,4008
IGHG4	Immunoglobulin heavy constant gamma 4	2.005	0.0494	1.0036	1.3060	0,1267
ORM1	Alpha-1-acid glycoprotein 1	2.017	0.0420	1.0122	1.3763	0,0039
VCL	Vinculin	2.021	0.0257	1.0150	1.5904	0,0678
SKP1	S-phase kinase-associated protein 1	2.027	0.0184	1.0193	1.7348	0,0552
LGALSL	Galectin-related protein	2.038	0.0345	1.0271	1.4620	0,2213
IL1RL1	Interleukin-1 receptor-like 1	2.038	0.0972	1.0271	1.0123	0,2515
GYG1	Glycogenin-1	2.039	0.0609	1.0278	1.2156	0,4088
ELOB	Elongin-B	2.056	0.0016	1.0398	2.7844	0,2199
RBP2	Retinol-binding protein 2	2.072	0.0041	1.0510	2.3866	0,2891
ARPC4	Actin-related protein 2/3 complex subunit 4	2.088	0.0749	1.0621	1.1252	0,1357
ASAP1	Arf-GAP with SH3 domain. ANK repeat and PH domain-containing protein 1	2.088	0.0821	1.0621	1.0855	0,1326
SH3BGRL3	SH3 domain-binding glutamic acid-rich-like protein 3	2.114	0.0480	1.0799	1.3187	0,0388
ACACA	Acetyl-CoA carboxylase 1	2.117	0.0909	1.0820	1.0415	0,0920
FABP1	Fatty acid-binding protein. liver	2.12	0.0105	1.0840	1.9794	0,0388
YWHAH	14-3-3 protein eta	2.138	0.0381	1.0962	1.4189	0,0399
ARHGDIB	Rho GDP-dissociation inhibitor 2	2.148	0.0553	1.1029	1.2573	0,0456
IGKJ1	Immunoglobulin kappa joining 1	2.175	0.0753	1.1210	1.1231	0,1011
GKN1	Gastrokine-1	2.195	0.0593	1.1342	1.2269	0,0596
S100A8	Protein S100-A8	2.214	0.0203	1.1466	1.6933	0,0014
S100P	Protein S100-P	2.219	0.0075	1.1499	2.1252	0,0942
ARPC2	Actin-related protein 2/3 complex subunit 2	2.237	0.0913	1.1615	1.0396	0,0943
PSMB2	Proteasome subunit beta type-2	2.288	0.0416	1.1940	1.3804	0,2305
MB	Myoglobin	2.289	0.0494	1.1947	1.3066	0,0109
SH3BGRL	Adapter SH3BGRL	2.311	0.0464	1.2085	1.3333	0,0812
ACTR2	Actin-related protein 2	2.391	0.0334	1.2576	1.4767	0,1206
ARPC1B	Actin-related protein 2/3 complex subunit 1B	2.467	0.0880	1.3027	1.0554	0,0257
S100A9	Protein S100-A9	2.527	0.0096	1.3374	2.0172	0,0009
FABP4	Fatty acid-binding protein. adipocyte	2.527	0.0348	1.3374	1.4589	0,0752
SLC25A31	ADP/ATP translocase 4	2.779	0.0187	1.4745	1.7281	0,1198
DBI	Acyl-CoA-binding protein	2.787	0.0103	1.4787	1.9862	0,0339
SLURP2	Secreted Ly-6/uPAR domain-containing protein 2	2.821	0.0015	1.4962	2.8221	0,0163
VASP	Vasodilator-stimulated phosphoprotein	3.099	0.0703	1.6318	1.1530	0,0054
S100A12	Protein S100-A12	3.108	0.0032	1.63598	2.4949	0,0000
TMSB4X	Thymosin beta-4	3.129	0.0723	1.6457	1.1407	0,0006
ILK	Integrin-linked protein kinase	3.191	0.0929	1.6740	1.0319	0,0006
C4A	Complement C4-A	3.285	0.0267	1.7158	1.5734	0,0049
MPIG6B	Megakaryocyte and platelet inhibitory receptor G6b	3.434	0.0379	1.7798	1.4214	0,0068
MYL1	Myosin light chain 1/3. skeletal muscle isoform	3.541	0.0018	1.8241	2.7390	0,0011
PGA4	Pepsin A-4	3.571	0.0260	1.8363	1.5844	0,0004
TUBB	Tubulin beta chain	3.758	0.0232	1.9099	1.6349	0,0000
CLTC	Clathrin heavy chain 1	3.783	0.0883	1.9195	1.0539	0,0046
CHGA	Chromogranin-A	3.881	0.0003	1.9564	3.5422	0,0000

MYL6B	Myosin light chain 6B	3.913	0.0416	1.9682	1.3811	0,0066
MAPRE2	Microtubule-associated protein RP/EB family member 2	4.17	0.0877	2.0600	1.0568	0,0001
HADHA	Trifunctional enzyme subunit alpha. mitochondrial	4.184	0.0165	2.0648	1.7834	0,0001
MMP3	Stromelysin-1	4.239	0.0152	2.0837	1.8178	0,0001
PPIF	Peptidyl-prolyl cis-trans isomerase F. mitochondrial	4.688	0.0679	2.2289	1.1684	0,0000
SAA1	Serum amyloid A-1 protein	4.825	0.0152	2.2705	1.8193	0,0000
CST1	Cystatin-SN	4.877	0.0815	2.2859	1.0887	0,0000
SEPTIN7	Septin-7	4.902	0.0216	2.2933	1.6652	0,0000
CLTA	Clathrin light chain A	4.985	0.0185	2.3175	1.7319	0,0001

Supplementary Table 4. Clinical features of patients included in the immunofluorescence study.

ID	Age	Sex	Class	Biopsy	Medication at the time of biopsy	Type of imaging
**1**	87	F	C	Negative	Nothing	PET negative at 3 months after starting therapy with GC
**2**	92	F	C	Negative	Nothing	None
**3**	85	F	C	Negative	Nothing	None
**4**	86	M	C	Negative	Prednisone 15 mg/d x 10 days	PET normal or negative two months after starting GC
**5**	82	M	PMR	Negative	Nothing	Thoracoabdominal CT normal
**6**	68	F	PMR	Negative	Prednisone 30 mg/d x 7 days	PET: negative at 6 months after starting therapy with GC
**7**	88	M	PMR	Negative	MTX 15 mg/week	None
**8**	75	F	PMR	Negative	Prednisone 1.25 mg/d	PET: Subdeltoid bursitis in both shoulders typical of PMR
**9**	69	F	EC-GCA	Negative	MPS 500 mg/iv only 1 day	PET negative at 5 months after starting therapy with GC
**10**	86	F	EC-GCA	Negative	Prednisone 60 mg/d x 5 days	None
**11**	64	F	EC-GCA	Negative	MPS 125 mg/d x 3 days	PET normal/negative
**12**	77	M	EC-GCA	Negative	Prednisone 40 mg/d x 10 days	PET negative, but done 9 months after starting therapy with GC
**13**	89	M	C-GCA	Positive	MPS 250 mg/iv only 1 day	Aortic & supra-aortic CT normal<br>PET negative/normal
**14**	86	F	C-GCA	Positive	Prednisone 60 mg/d x 3 days	None
**15**	81	F	C-GCA	Positive	Nothing	PET/CT negative at 2 months after TAB
**16**	77	F	C-GCA	Positive	Prednisone 50 mg/d x 2 day	PET<br>Periarticular hypercaptation in shoulders and spine, two years later

ID: identifier; class: classification; F: female; M: male; C: control; PMR: polymyalgia rheumatica; EC-GCA: extracranial GCA; C-GCA: cranial GCA; IVIG: intravenous immunoglobulins; MPS: methylprednisolone intravenous boluses; MTX: methotrexate; RTX: rituximab; TAB: temporal artery biopsy.
